# Molecular Characterization of Plant Growth-Promoting Bacteria Associated with *Opuntia dillenii* (Ker Gawl.) Haw (Cactaceae) in the Coastal Zone of Benin

**DOI:** 10.3390/microorganisms14061376

**Published:** 2026-06-21

**Authors:** Yves Kévin Brun, Agossou Damien Pacôme Noumavo, Julien Colombet, Etienne Bankolé Atchadé, Lamine Baba-Moussa, François Lefort

**Affiliations:** 1 Plants and Pathogens Group, Research Institute Land Nature Landscape, HEPIA Geneva School of Engineering Architecture and Landscape, HES-SO University of Applied Sciences and Arts Western Switzerland, 150 Route de Presinge, 1254 Jussy, Switzerland; kevin.brun@etu.hesge.ch (Y.K.B.); julien.colombet@hesge.ch (J.C.); 2 Laboratory of Microbiology, Food Technology and Phytopathology, Faculty of Science and Technology, University of Abomey-Calavi, Abomey-Calavi 05 P.O. Box 1604, Benin; 3 Laboratory of Biology and Molecular Typing in Microbiology, Faculty of Science and Technology, University of Abomey-Calavi, Cotonou 04 P.O. Box 1107, Benin; laminesaid@yahoo.fr; 4 Laboratory of Soil, Water and Environment Sciences, National Institute of Agricultural Research of Benin, Cotonou 01 P.O. Box 884, Benin; benatchade@gmail.com

**Keywords:** *Opuntia dillenii*, rhizobacteria, endophytes, coastal microbial resources, plant growth-promoting bacteria, 16S rRNA, *gyrB*, *Priestia flexa*, Benin

## Abstract

Cacti thrive in arid and coastal environments partly through associations with beneficial endophytic and rhizosphere bacteria; however, current knowledge remains limited. This study aimed to assess the diversity of cultivable bacteria associated with *Opuntia dillenii* and evaluate their potential as Plant Growth-Promoting (PGP) agents. Endophytic bacteria were isolated from cladodes and roots, while rhizobacteria were recovered from rhizosphere soil. Bacterial isolates were identified using morphological characteristics and 16S rRNA/gyrB sequencing, followed by screening for PGP traits, pH and temperature tolerance. A total of 31 isolates were obtained, including 23 endophytes and 8 rhizobacteria, mainly affiliated with Firmicutes, Actinobacteria, and Proteobacteria. *Bacillus* (35.48%) and *Priestia* (32.25%) predominated, with *Priestia flexa* as the most prevalent species. The most frequent PGP traits were phosphate solubilization (80.65%), proteolytic activity (70.97%), siderophore production (67.74%), and nitrogenase activity (64.52%). The highest phosphate solubilization indices were observed for strain R3 (3.41), R6 (3.39) and S6 (3.21), whilst the highest indole-3-acetic acid yields were recorded for C9 (172.88 µg/mL), R11 (96.22 µg/mL) and C3 (90.94 µg/mL), and the strongest siderophore production for C3 (30.37 mm), C7 (27.96 mm) and S7 (27.88 mm). These findings highlight *O. dillenii*-associated coastal bacteria as promising resources for plant growth and plant stress resilience.

## 1. Introduction

Currently estimated at 8.2 billion, the world’s population is projected to reach 9.7 billion by 2050 [1]. This growth would lead to a 59% to 98% increase in food demand [2]. Consequently, it will be necessary to significantly increase agricultural production in the coming years. There are two main strategies: expanding the amount of arable land or boosting the productivity of existing farmland. The latter option largely depends on the intensive application of agrochemical inputs such as fertilizers, pesticides, and herbicides. Excessive use of agrochemical inputs impairs soil health by altering its physicochemical properties including pH, salinity, nutrient availability, structure, porosity, and compaction as well as its biological properties, such as enzymatic activity and the composition of the soil microbiome.

In recent years, increasing attention has focused on sustainable and environmentally sound alternatives, particularly those based on beneficial plant–microbe interactions. Among these, plant growth-promoting bacteria (PGPB) represents a functionally defined group of bacteria capable of enhancing plant growth and resilience to biotic and abiotic stresses [3]. PGPB promote plant growth through a broad range of direct and indirect mechanisms, including biological nitrogen fixation, solubilization of mineral nutrients (phosphorus, potassium, and iron), phytohormone production, siderophore synthesis, ACC deaminase activity, suppression of phytopathogens, and induction of systemic resistance in plants [4,5]. From an ecological perspective, PGPB are classified according to their colonization niche into two major compartments: the rhizosphere and the endosphere.

The rhizosphere, first defined by Hiltner, is the narrow zone of soil surrounding plant roots that is directly influenced by root activity and characterized by intense microbial processes [6]. This microhabitat supports dense and metabolically active microbial communities distinct from those of bulk soil [7]. It provides an ideal microhabitat for many beneficial bacteria known as plant growth-promoting rhizobacteria (PGPR).

In contrast to the rhizosphere, a plant’s endosphere comprises all the internal tissues colonized by microorganisms, particularly bacteria, fungi and archaea [8]. Endophytic bacteria inhabit plant tissues without causing harm to the host [9] and may colonize roots, stems, leaves, seeds, and flowers [10]. These endophytes contribute to plant growth, enhance tolerance to abiotic stresses such as drought and salinity, and improve resistance to pathogens [11].

Together, rhizosphere and endophytic bacterial communities form close associations with their host plants that may range from mutualistic to commensal or, less frequently, parasitic [12]. Bacteria belonging to the genera *Bacillus*, *Azoarcus*, *Pseudomonas*, *Serratia*, *Enterobacter*, *Streptomyces*, *Herbaspirillum*, *Azospirillum*, *Azotobacter*, *Klebsiella*, *Alcaligenes*, *Arthrobacter*, *Burkholderia*, *Microbacterium*, *Micrococcus*, *Pantoea*, and *Stenotrophomonas* [7,13,14,15,16,17,18,19,20].

Plants adapted to extreme or resource-limited environments are increasingly recognized as reservoirs of functionally specialized microbial communities. Cacti (Cactaceae) are renowned for their ability to thrive in nutrient-poor soils and withstand extreme climatic conditions, making them ideal hosts for diverse microbial communities. The genus *Opuntia* comprises between 200 and 300 species [21]. Among these, *Opuntia dillenii* is one of the dominant plant species in the coastal zone of Benin, a transitional ecosystem between terrestrial and marine environments, characterized by sandy soils with low nutrient content, variable salinity, marked seasonal drought and high anthropogenic pressure [22]. In this challenging environment, xerophytic and halotolerant plant species survive and thrive thanks to remarkable ecological adaptation mechanisms.

Despite the ecological importance and adaptive potential of these plant–microbe interactions, the microbiome associated with *Opuntia dillenii* in coastal environments remains largely uncharacterized. Yet, microorganisms inhabiting plants adapted to saline and arid conditions are assumed to possess unique functional traits related to plant growth promotion and stress tolerance, which could be exploited for sustainable agricultural applications. In this context, the present study is part of a bioprospecting approach aimed at valorizing endophytic and rhizosphere bacteria as biological resources for improving plant health and promoting environmentally friendly agricultural practices. Specifically, this study aims to assess the diversity and plant growth-promoting (PGP) potential of cultivable endophytic and rhizosphere bacterial strains associated with *O. dillenii* in the coastal zone of Benin. By generating new insights into locally adapted beneficial bacteria, this work seeks to contribute to a better understanding of their role in enhancing plant resilience and productivity in underexplored agroecological zones and to support their potential use in the development of sustainable bio-inoculants.

## 2. Materials and Methods

### 2.1. Isolation of Cultivable Endophytic and Rhizosphere Bacteria from O. dillenii

#### 2.1.1. Sampling of *O. dillenii* and Rhizosphere Soil

Composite sampling was conducted to obtain a representative estimate of the local microbial community [23]. To this end, samples of cladodes, roots, and rhizosphere soil were collected from three healthy *O. dillenii* plants and placed in sterile polyethylene bags in December 2024 in Togbin (6°22′24″ N, 2°18′32″ E), in the coastal zone of Benin. After labeling, the samples were transported to the laboratory in a cooler at approximately 4 °C. Upon arrival, the isolation of cultivable endophytic and rhizosphere bacteria was performed immediately.

#### 2.1.2. Surface Sterilization of *O. dillenii* Cladodes and Roots

The cladodes and roots of *O. dillenii* were subjected to surface sterilization to eliminate unwanted dirt’s and surface-adhering microorganisms, following the method described by Ratnaweera et al. [24], with slight modifications. The roots, which had accumulated substantial soil and debris, were first washed under running water for 10–15 min, while the cladodes were washed for 10 min. The samples were then immersed sequentially in 70% ethanol (1–2 min), in a 3% sodium hypochlorite solution (5 min), and again in 70% ethanol (30–60 s). Finally, each plant organ was rinsed three times with sterile distilled water, for 1 min per rinse, to remove residual disinfectants. The sterilized tissues were then left to dry on sterile blotting paper inside a laminar flow hood.

#### 2.1.3. Isolation of Cultivable Endophytic Bacteria

This step consisted of inoculating the previously sterilized samples onto Nutrient Agar (NA) (HIMEDIA, Thane, India). The samples were first carefully cut into small fragments of approximately 1 cm^2^ on sterile blotting paper using sterile forceps and a scalpel under aseptic conditions. Seven fragments from each plant organ were then placed in Petri dishes containing NA and incubated for 48 h at 28 ± 2 °C. To verify the effectiveness of surface sterilization, 100 µL of the final rinse water was spread onto NA as a control [25]. Colonization frequency was calculated as the percentage of plant segments showing bacterial growth relative to the total number of incubated segments.

#### 2.1.4. Isolation of Cultivable Rhizosphere Bacteria

Bacteria were isolated on NA after decimal dilution of rhizosphere soil following the method described by Speck [26]. 10 g of rhizosphere soil were aseptically transferred to 90 mL of sterile distilled water (SDW) in a 250 mL conical flask to prepare the stock suspension. Decimal dilutions were then performed up to 10^−5^. Aliquots of 0.1 mL from the 10^−3^, 10^−4^, and 10^−5^ dilutions were then spread, in triplicate, onto NA plates. The plates were incubated at 28 ± 2 °C for 24 h. The bacterial load of the rhizosphere soil was determined according to ISO 7218:2024 [27] using the weighted mean method. The concentration (N) in CFU·g^−1^ was calculated using the formula

(1) $$N=\frac{\sum C\;}{V\;\times 1.1\times \;d}$$

where ∑C is the sum of colonies counted on the two selected plates, V is the inoculum volume (0.1 mL), and d is the dilution factor of the lowest selected dilution [28].

#### 2.1.5. Purification and Storage of Bacterial Isolates

The previously isolated and morphologically distinct cultivable rhizosphere and endophytic bacterial strains were purified according to the method described by Fasusi et al. [29]. The colonies were subcultured several times on NA until pure cultures were obtained. The purified strains were then stored at −20 °C in Nutrient Broth (HIMEDIA) supplemented with 30% glycerol.

### 2.2. Physicochemical Analyses of Rhizosphere Soil

The texture of the rhizosphere soil was determined using the Robinson-Kôhn pipette method, based on Stokes’ law [30]. Total nitrogen (N) was determined using the Kjeldahl method [31] with a TitroLine^®^ 5000 analyzer (SI Analytics, Mainz, Germany). Organic carbon (C) was measured using the Walkley & Black method, using potassium dichromate [32]. Soil organic matter (OM) was determined using the Loss On Ignition (LOI) method, measuring the loss of mass after drying at 220 °C, then calcination at 550 °C,

(2) $$\mathrm{LOI}\;(\%)=\frac{M_{1}-M_{2}}{M_{1}-M_{0}}\times 100$$

where *M*_0_ = mass of empty crucible (g), *M*_1_ = mass of crucible + soil after drying at 220 °C (g), and *M_2_* = mass of crucible + ashes after ignition at 550 °C (g). Assimilable phosphorus (P) was evaluated using Bray I acid solution, followed by colorimetric titration with ammonium molybdate blue, with a reading at 600 nm [33]. Exchangeable cations (Na^+^, K^+^, Ca^2+^, Mg^2+^) were determined using the Schollenberger method, by leaching 2.5 g of soil with 25 mL of 1 M ammonium acetate buffered to pH 7 [34]. The cation exchange capacity (CEC) was obtained using the ammonium–KCl method, which consists of saturating the exchange sites with NH_4_^+^ and then displacing it with a KCl solution, The leaching operation was repeated three times to ensure complete extraction of the fixed NH_4_^+^, in accordance with the standard protocols ISO/TS 22171:2023 [35]. The pH of the soil was measured using a potentiometric method on a soil–water suspension at a ratio of 1:2.5. After stirring and decanting, the reading was taken using a calibrated pH meter [36]. Electrical Conductivity (EC), salinity, and Total Dissolved Solids (TDS) were measured simultaneously using a multiparameter probe on a soil–water suspension (1:5) that was stirred and then decanted, according to the FAO protocol [37] described in the Global Soil Laboratory Network (GLOSOLAN) procedures.

### 2.3. Morphological Characterization of Bacterial Isolates

The bacterial isolates were seeded on NA poured into Petri dishes and incubated at 28 ± 2 °C for 24 h. After incubation, the plate morphological characteristics, such as shape, size, surface, elevation, opacity, color, consistency, and margin were observed and recorded.

Each bacterial strain was then subjected to Gram staining, according to the protocol described by Riegel et al. [38]. Microscopic observation was used to determine the Gram type (positive or negative), cell shape (coccus or rod), and cell grouping pattern.

### 2.4. Molecular Characterization of the Bacterial Isolates

#### 2.4.1. DNA Extraction

Total DNA was extracted from purified colonies using a CTAB (hexadecyltrimethylammonium bromide) protocol adapted from Ripoll et al. [39]. Each bacterial isolate was cultured in Luria–Bertani broth (LBB, Roth AG, Arlesheim, Switzerland) for 24 h at 30 °C with constant agitation. The bacterial pellets were recovered by centrifugation at 8000 rpm for 5 min, then lysed in CTAB buffer preheated to 65 °C, followed by extraction with chloroform/isoamyl alcohol (24:1). DNA was precipitated with isopropanol, washed with 70% ethanol, and re-suspended in sterile ultrapure water. The concentration and purity of the extracted DNA were assessed using a NanoDrop ND-1000 spectrophotometer (Thermo Fisher Scientific, Geneva, Switzerland).

#### 2.4.2. PCR Amplification of 16S rDNA and *GyrB* Genes

The 16S rDNA and *gyrB* genes were amplified by PCR from bacterial DNA in a 25 µL reaction volume using a SimpliAmp thermocycler (Thermo Fisher Scientific) and MyTaq™ DNA polymerase (LabGene Scientific, Châtel-Saint-Denis, Switzerland) in accordance with the manufacturer’s guidelines. Amplification of the 16S rDNA gene was performed using primers 27F (5′-AGA GTT TGA TCC TGG CTC AG-3′) [40] and 1492R (5′-GGT TAC CTT GTT ACG ACT T-3′) [41], while the *gyrB* gene was amplified using the primers UP1 (5′-GAA GTC ATC ATG ACC GTT CTG CAY GCN GGN GGN AAR TTY GA-3′) and UP2R (5′-AGC AGG GTA CGG ATG TGC GAG CCR TCN ACR TCN GCR TCN GTC AT-3′) [42]. The PCR conditions for amplification process is presented in Table 1 and Table 2. The amplification products of the two genes were separated by electrophoresis on a 1.5% (*w*/*v*) agarose gel (Roth AG) in 1X TBE buffer (Roth AG), stained with GelRed (Biotium; Roth AG), and visualized using a U: Genius 3 transilluminator (LabGene Scientific).

#### 2.4.3. Sequencing

After purification of the PCR products using the Wizard^®^ SV Gel and PCR Clean-Up System Kit (Promega, Dübendorf, Switzerland), the amplicons were subjected to Sanger sequencing at Microsynth (Balgach, Switzerland). The chromatograms obtained were viewed and edited using FinchTV v.1.5.0. The consensus sequences were then compared to those available in the NCBI (National Center for Biotechnology Information, Bethesda, MA, USA) nucleotide database using the BLASTn tool [43]. The phylogenetic relationship between the bacterial isolates was analyzed using Mega 12.1 software (https://www.megasoftware.net), employing the Maximum Likelihood method and the General Time Reversibility model.

### 2.5. In Vitro Screening of Plant Growth-Promoting (PGP) Traits and Extracellular Enzyme Activities

To assess the different plant growth-promoting (PGP) traits, the bacterial inoculum was prepared by suspending a colony in 10 mL of LB broth and incubating the suspension at 30 °C under shaking at 150 rpm until the exponential growth phase was reached (OD_600_ ≈ 0.6). Each experiment was carried out in triplicate.

#### 2.5.1. Production of Indole-3-Acetic Acid (IAA)

IAA production was evaluated in the presence of L-tryptophan using the method described by Gordon and Weber [44], with some modifications. The bacterial strains were cultured in 20 mL of LB broth supplemented with L-tryptophan (100 mg/L), then incubated at 28–30 °C for 48 h with agitation (150 rpm). After incubation, the cultures were centrifuged at 6000 rpm for 10 min to recover the supernatant. One milliliter of this supernatant was then mixed with 2 mL of Salkowski’s reagent (15 mL FeCl_3_ 0.5 mol/L, 300 mL H_2_SO_4_, 500 mL distilled water) [45], and the mixture was incubated in the dark. Following a 30 min incubation, the optical density was measured at 530 nm using a UV spectrophotometer (Thermo Fisher Scientific). For quantification, a standard curve was generated from a stock solution of IAA (1000 µg/mL), which was diluted to obtain standard concentrations between 0 and 100 µg/mL. The absorbance of the samples at 530 nm was then converted into IAA concentration using this calibration curve. The development of a pink color indicated the production of IAA.

#### 2.5.2. Siderophore Production

Siderophores production by bacterial strains was detected on Chrome Azurol S (CAS) agar, following the modified protocol of Schwyn and Neilands [46]. First, 900 mL of Luria–Bertani Agar (LBA, Roth AG, Arlesheim, Switzerland) was prepared by dissolving 5 g of yeast extract, 10 g of NaCl, and 15 g of agar in deionized water, then sterilized in an autoclave. At the same time, the CAS reagent was prepared by dissolving 72.9 mg of hexadecyltrimethylammonium bromide (HDTMA) in 40 mL of deionized water and 60.5 mg of CAS dye in 50 mL of deionized water, each in separate 100 mL beakers. A fresh 1 mM iron (III) chloride stock solution was prepared, and 10 mL of this was added to the HDTMA-CAS mixture to complete the CAS reagent preparation. The final volume was adjusted to 100 mL. After cooling to approximately 45–50 °C, the LBA and the CAS reagent were aseptically mixed with at 1:10 ratio (100 mL of CAS reagent per 900 mL of LBA). Sterile 6 mm diameter Whatman filter paper discs (ROTILABO, Karlsruhe, Germany) were aseptically placed on the surface of the solidified medium in Petri dishes. Ten microliters (10 µL) of the bacterial inoculum were inoculated directly onto the filter paper discs, and the plates were incubated at 30 °C for 72 h. Siderophore production was indicated by the appearance of a yellow-orange halo around the colonies.

#### 2.5.3. Atmospheric Nitrogen Fixation

The atmospheric nitrogen fixation capacity of the bacterial strains was evaluated on Jensen medium [47], composed of 20 g of sucrose, 0.5 g of NaCl, 0.5 g of MgSO_4_·7H_2_O, 1.0 g of K_2_HPO_4_, 0.005 g of Na_2_MoO_4_, 2.0 g of CaCO_3_, 0.1 g of FeSO_4_·7H_2_O, and 15 g of agar per 1000 mL of sterile distilled water, adjusted to a pH of 7.2. Sterile 6 mm diameter Whatman filter paper discs (ROTILABO, Germany) were aseptically placed on the surface of the solidified medium in Petri dishes. Ten microliters of the bacterial inoculum were inoculated directly onto the filter paper discs, and the dishes were incubated at 30 °C for 72 h. Bacterial growth on the medium indicated a positive capacity for atmospheric nitrogen fixation [48].

#### 2.5.4. Phosphate Solubilization

Phosphate solubilization by bacterial strains was assessed using the National Botanical Research Institute’s Phosphate (NBRIP) medium (10 g glucose, 5 g Ca_3_(PO_4_)_2_, 5 g MgCl_2_·6H_2_O, 0.25 g MgSO_4_·7H_2_O, 0.2 g KCl, 0.1 g (NH_4_)_2_SO_4_, 15 g agar per 1000 mL distilled water, pH 7.0) [49]. Sterile 6 mm diameter Whatman filter paper discs (ROTILABO, Germany) were aseptically placed on the surface of the solidified medium in Petri dishes. Ten microliters of the bacterial inoculum were inoculated directly onto the filter paper discs, and the dishes were incubated at 30 °C for 72 h. The appearance of a clear halo around the discs indicated positive phosphate solubilization activity, as described by Carvalhais and Dennis [49]. An uninoculated Petri dish served as a control. The diameter of each halo (Dh) as well as that of the corresponding colony (Dc) were measured for each bacterial isolate. These measurements were then used to calculate the phosphate solubilization index (PSI), according to the formula: PSI = Dh/Dc.

#### 2.5.5. Exopolysaccharide Production

Bacterial strains exopolysaccharide production was assessed on LB agar medium supplemented with 10% sucrose, adjusted to a pH of 7.0, and then sterilized at 121 °C for 15 min. Sterile 6 mm diameter Whatman filter paper discs (ROTILABO, Germany) were aseptically placed on the surface of the solidified medium in Petri dishes. Ten microliters of the bacterial inoculum were inoculated directly onto the filter paper discs, and the dishes were incubated at 30 °C for 24 h. The appearance of a mucoid texture on the filter paper discs indicated a positive result for the production of exopolysaccharides [48].

#### 2.5.6. Lipolytic Activity

Lipolytic activity was assessed on a Tween 80 agar medium composed of (g/L) peptone (10 g), yeast extract (5 g), NaCl (5 g), agar (20 g), and 10 mL of Tween 80, adjusted to a pH of 7.5 and then sterilized at 121 °C for 15 min. Sterile 6 mm diameter Whatman filter paper discs (ROTILABO, Germany) were aseptically placed on the surface of the solidified medium in Petri dishes. Ten microliters of the bacterial inoculum were inoculated directly onto the filter paper discs, and the dishes were incubated at 30 °C for 48 h. The appearance of clear zones around the colonies indicated the hydrolysis of Tween 80 and thus positive lipolytic activity. The diameter of these halos was used as an indicator of the intensity of the enzymatic activity produced [50]. An uninoculated Petri dish served as a control.

#### 2.5.7. Proteolytic Activity

Proteolytic activity was assessed using the adapted method of Dogan and Taskin [51] with LB agar supplemented with 1% (*w*/*v*) skim milk powder. To prepare this supplement, 10 g of skim milk powder were dissolved in 100 mL of distilled water, then sterilized at 110 °C for 5 min. The milk solution was then cooled to 45 °C before being aseptically added to the sterile LBA to obtain a homogeneous medium. The prepared medium was distributed into Petri dishes. Sterile 6 mm diameter Whatman filter paper discs (ROTILABO, Germany) were aseptically placed at the surface of the solidified medium in Petri dishes. Ten microliters of bacterial inoculum were then placed on each disc, and the dishes were incubated at 30 °C for 48 h. The appearance of a clear zone indicative of positive proteolytic activity.

#### 2.5.8. Amylolytic Activity

Amylolytic activity was assessed on NA supplemented with 1% soluble starch (10 g/L). The strains were streaked onto the surface of the medium and incubated at 30 °C for 24 h. After incubation, plates were flooded with Lugol’s solution (0.2% iodine and 0.4% potassium iodide in 100 mL of distilled water). The presence of amylolytic activity was indicated by the appearance of zones of clarification around the bacterial streaks, indicating starch hydrolysis [52].

#### 2.5.9. Additional Screening

All strains were subjected to growth tests at different pH levels and temperatures to determine their range of resilience to abiotic conditions unfavorable to plant growth. Specifically, strains were cultured on NA adjusted to pH values of 5, 6, 7, 8, 9, 10, and 11 using 0.1 M HCl or 0.1 M NaOH, and incubated at different temperatures: 25, 30, 35, 40, 45, 50, and 55 °C [53]. The plates were incubated at 30 °C for 48 h. Growth performance under different pH and temperature conditions were evaluated using the following scale: (+): normal growth, (++): abundant growth and (-): no growth.

### 2.6. Statistical Analyses

Statistical analysis was performed using R (version 4.4.3). Taxonomic α-diversity indices, including species richness (S), Shannon index (H′), Simpson index (1 − D), and Pielou’s evenness (J′), were calculated using the vegan package (version 2.7-3) in R [54]. One-way analysis of variance (ANOVA) was conducted to assess significant differences between strains. In cases of significant differences (*p* < 0.05), means were compared using the Student–Newman–Keuls (SNK) post hoc test. Principal component analysis (PCA) was performed using the FactoMineR package (version 2.13) in R after data standardization, with component retention based on Kaiser’s criterion (eigenvalues > 1) [55]. Statistical associations between variables and PCA axes were assessed using dimdesc [56]. Hierarchical clustering on principal components (HCPC) was applied to identify functional groups among isolates. Graphical representations were generated using the factoextra package (version 1.0.7). The ggplot2 package (version 4.0.1) was used to create the heatmaps.

## 3. Results

### 3.1. Colonization Rate and Isolation Rate of Bacterial Isolates

Out of the 84 plant segments, 58 showed bacterial growth, representing an overall colonization rate of 69.04%. Colonization was higher in the roots (83.33%) than in the cladodes (54.76%). Twenty-three morphologically distinct cultivable endophytic bacteria were isolated from *O. dillenii*, 12 isolates from cladodes and 11 from roots, yielding a total isolation rate of 27.38%. In addition, eight cultivable rhizobacteria were isolated from the rhizosphere of *O. dillenii*, with an average density of 4.3 × 10^5^ CFU·g^−1^.

### 3.2. Physicochemical Composition of Rhizosphere Soil

The granulometric analysis indicated that the rhizosphere soil was almost exclusively sandy, resulting in high permeability to water and nutrients. From a chemical standpoint, the soil was characterized by very low fertility, with minimal organic matter and nitrogen contents, reflecting a weakly humified and easily biodegradable organic fraction. The slightly acidic pH further reflects the poor buffering capacity of the soil. Exchangeable cations and cation exchange capacity were extremely low, consistent with the limited charge and retention capacity of the sandy soil matrix, despite a high base saturation. Phosphorus availability was also low. In addition, salinity, total dissolved solids, and electrical conductivity values indicated a weakly mineralized soil solution (Table 3). Overall, these physicochemical characteristics describe a highly oligotrophic coastal soil, imposing strong edaphic constraints on microbial communities associated with *Opuntia dillenii*.

### 3.3. Morphological Diversity of Bacterial Isolates

The bacterial colonies were predominantly circular (77.42%), flat (67.74%), medium-sized (58.06%), smooth-surfaced (64.52%), and opaque (80.65%). Most isolates exhibited a creamy consistency (64.52%), although some displayed dry or mucoid textures. A minority of strains produced yellow pigmentation on nutrient agar (12.90%). The community was largely composed of Gram-positive bacteria (80.65%), with an overwhelming predominance of rod-shaped cells (93.55%). Detailed macroscopic and microscopic characteristics of all 31 isolates are presented in Table 4, Table 5 and Table 6.

### 3.4. Phylogeny Based on the 16S rRNA Gene of Bacteria Associated with O. dillenii

**Figure 1:**
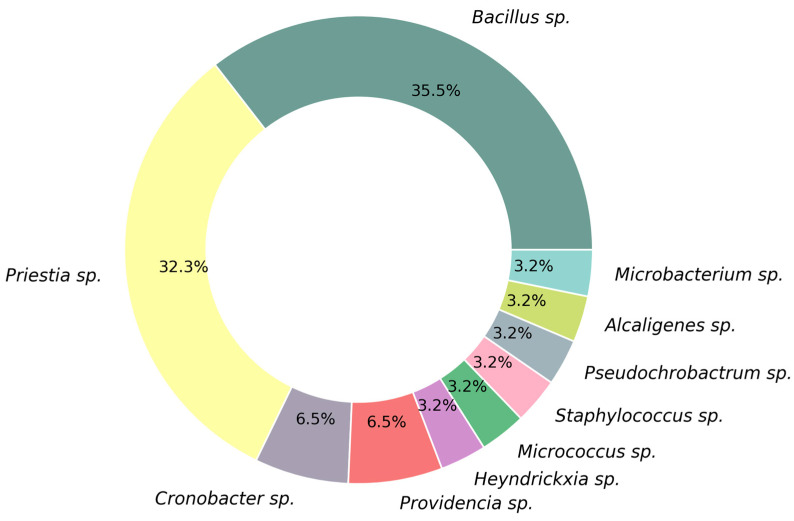
Proportion of bacterial genera in *O. dillenii*.

**Figure 2:**
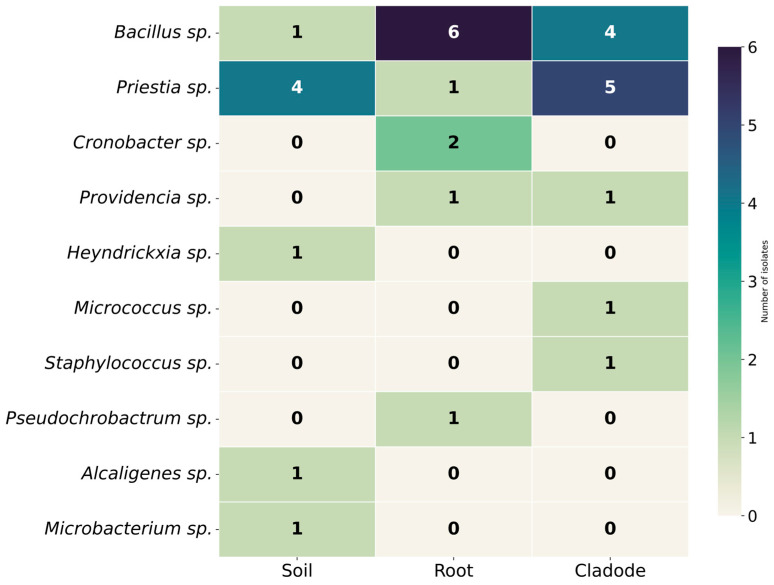
Abundance profile of bacterial genera according to the organs of *O. dillenii*.

The 16S rRNA gene sequences obtained were deposited in the NCBI Nucleotide database (https://www.ncbi.nlm.nih.gov/nucleotide/) (accessed on 5 March 2026) under the accession numbers PZ102195–PZ102225 (Table 7). Sequence comparison with reference entries in the NCBI database revealed high identity values ranging from 99.40% to 100%, confirming the reliability of the taxonomic assignments. The isolates mainly belonged to the phyla Firmicutes, Actinobacteria, and Proteobacteria. At the genus level, the cultivable bacterial community was dominated by *Bacillus* (35.48%) and *Priestia* (32.25%) (Figure 1). The distribution of these genera varied across plant compartments, with *Bacillus* spp. predominating in roots (54.54%), *Priestia* spp. in cladodes (41.66%), and *Priestia* spp. also dominating the rhizosphere soil (50%) (Figure 2). Although species richness was identical among compartments, marked differences in community structure were observed. Rhizosphere soil exhibited the highest diversity and evenness, followed by cladodes, whereas roots showed the lowest values. Simpson’s diversity index further supported slightly higher diversity in soil and cladode compartments compared with roots (Table 8). Overall, ten bacterial genera were identified, with community structure characterized by relatively high diversity but reduced evenness due to the strong dominance of *Bacillus* and *Priestia*.

**Figure 3:**
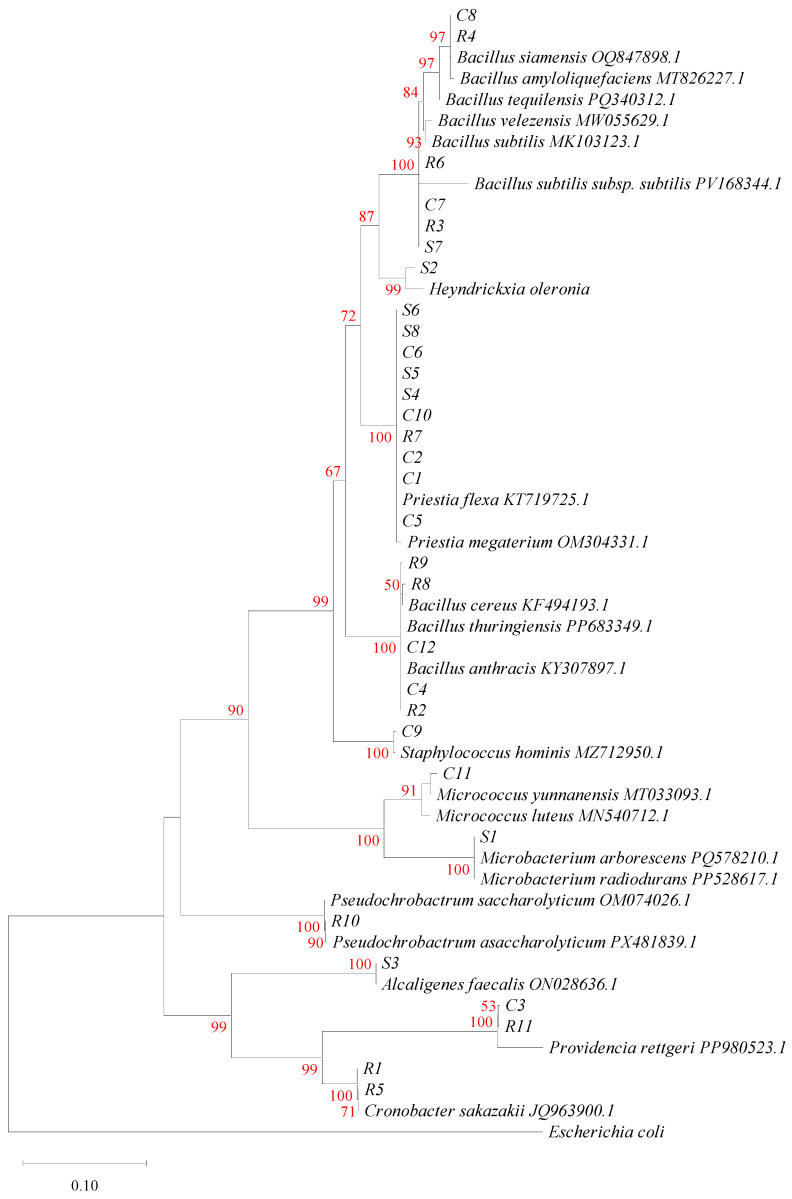
Phylogenetic tree (Maximum Likelihood, PhyML) based on the 16S rRNA sequences of bacterial isolates. Bootstrap = 1000 replications.

Among the isolates affiliated with the genus *Bacillus*, several belonged to the *Bacillus subtilis* sensu lato complex (C7, R3, R6, S7, C8, and R4), while others were assigned to the *Bacillus cereus* complex (C4, C12, R2, R8, and R9). Within the genus *Priestia*, *Priestia flexa* was the predominant species (32.25%) and was assigned to multiple isolates (S4, S5, S6, S8, C1, C2, C5, C6, C10, and R7), all showing 100% sequence identity with reference 16S rRNA gene sequences. Isolate S6 exhibited 100% identity with both *P. flexa* and *P. megaterium*, highlighting the close phylogenetic relationship between these two species and the limited resolving power of the 16S rRNA marker at the species level. Additional bacterial taxa were identified, including *Providencia rettgeri* (C3, R11) and *Cronobacter sakazakii* (R1, R5), as well as several less abundant species such as *Microbacterium aborescens* (S1), *Micrococcus yunnanensis* (C11), *Alcaligenes faecalis* (S3), *Heyndrickxia oleronia* (S2), *Staphylococcus hominis* (C9), and *Pseudochrobactrum asaccharolyticum* (R10) (Figure 3).

### 3.5. Phylogeny Based on the gyrB Gene of Bacteria Associated with O. dillenii

**Figure 4:**
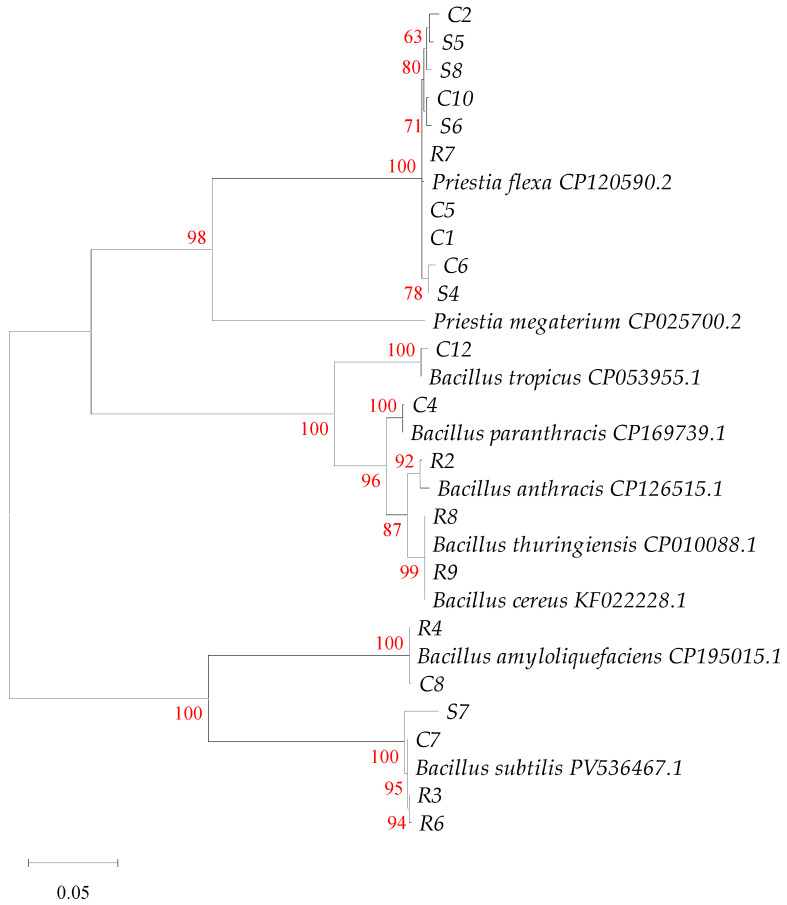
Phylogenetic tree (Maximum Likelihood, PhyML) based on *gyrB* sequences from bacterial isolates. Bootstrap = 1000 replications.

*GyrB* gene sequencing was performed for isolates that showed ambiguous taxonomic identification based on 16S rRNA gene sequences, particularly those affiliated with the genera *Priestia* and *Bacillus*. The *gyrB* sequences obtained were deposited in the NCBI Nucleotide database (https://www.ncbi.nlm.nih.gov/nucleotide/) (accessed on 23 March 2026) under the accession numbers PZ149653–PZ149673 (Table 9). Phylogenetic analysis based on the *gyrB* marker confirmed the specific affiliation of most isolates. Isolates S6, R7, C1, C2, C4, C5, C6, C10, S4, and S5 clustered with *Priestia flexa* (CP120590.2), confirming that isolate S6 belongs to this species and can be reliably distinguished from *P. megaterium*, a discrimination that was not possible using the 16S rRNA gene alone. Isolates belonging to the *Bacillus subtilis* sensu lato complex were resolved into two distinct groups: isolates C7, R3, R6, and S7 were clearly affiliated with *Bacillus subtilis* (CP174496.1), whereas isolates C8 and R4 clustered with *Bacillus amyloliquefaciens* (CP195015.1), confirming their species-level identification. In contrast, isolates R8 and R9, associated with the *Bacillus cereus* sensu lato complex, clustered within a clade comprising mainly *B. cereus* (KF022228.1) and *B. thuringiensis* (CP010088.1), two phylogenetically closely related species, together with other affiliated taxa such as *B. anthracis* (CP126515.1) and *B. paranthracis* (CP169739.1) (Figure 4).

### 3.6. PGP and Enzymatic Potentials of Bacterial Strains

#### 3.6.1. Indole-3-Acetic Acid (IAA) Production

**Figure 5:**
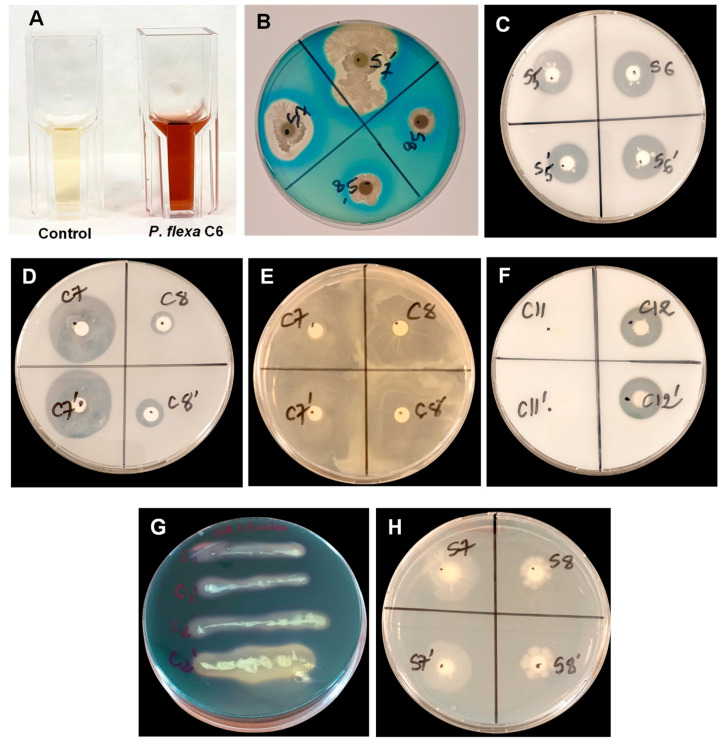
PGP traits of bacterial strains. Legend: (**A**) IAA biosynthesis in *P. flexa* C6; (**B**) Siderophore production by *B. subtilis* S7 and *P. flexa* S8; (**C**) Phosphatase activity of *P. flexa S5* and *P. flexa* S6; (**D**) Nitrogenase activity of *B. subtilis* C7 and *B. amyloliquefaciens* C8; (**E**) EPS activity of *B. subtilis* C7 and *B. amyloliquefaciens* C8; (**F**) Proteolytic activity of *B. tropicus* C12; (**G**) Amylolytic activity of *P. flexa* C1 and C2; (**H**) Lipase activity of *B. subtilis* S7.

In total, 38.71% of the bacterial strains produce IAA. This production varies highly significantly among strains (one-way ANOVA: F = 2138; df = 30 and 62; *p* < 2 × 10^−16^). This trait is observed mainly among isolates from cladodes: 83.33% of them produce IAA, with notably high concentrations found in *S. hominis* C9 (172.88 µg/mL), *P. rettgeri* C3 (90.94 µg/mL), and *P. flexa* C6 (71.94 µg/mL). In contrast, only one root isolate (*P. rettgeri* R11) produced IAA (96.22 µg/mL). A similar pattern was seen in soil, where only *M. aborescens* S1 exhibited IAA production (33.05 µg/mL) (Table 10, Figure 5). Consequently, cladode isolates represent the vast majority of IAA-producing strains in this collection.

#### 3.6.2. Siderophore Production

Siderophore production was detected in 67.74% of the bacterial strains, with highly significant differences between strains (one-way ANOVA: F = 95.7; df = 30 and 62; *p* < 2 × 10^−16^). This activity was observed in all three compartments studied, but with variable distribution depending on the origin of the strains. Among the cladode isolates, several strains exhibit high levels of production, notably *P. rettgeri* C3 (30.37 mm) and *B. subtilis* C7 (27.96 mm). At the root level, this trait is also well represented, with notable values in *P. rettgeri* R11 (26.92 mm), *B. anthracis* R2 (26.45 mm), and *B. amyloliquefaciens* R4 (24.74 mm). In the soil, *B. subtilis* S7 also stands out for its high activity (27.88 mm) (Figure 5 and Table 10). Overall, siderophore production is a widely distributed trait within this bacterial collection, with more consistent expression in root-associated strains (90.91%).

#### 3.6.3. Atmospheric Nitrogen Fixation

The ability to fix atmospheric nitrogen was observed in 64.52% of the bacterial strains studied. It was found in all three source compartments, with a higher prevalence among cladode isolates (75%). The highest expression levels were recorded in *B. subtilis* C7 for cladode isolates, in *B. subtilis* R3 and *C. sakazakii* R5 for roots, and in *P. flexa* (S4, S5, and S6) in the soil (Figure 5 and Table 10). These results indicate that biological fixation of atmospheric nitrogen is a trait widely shared within the bacterial collection studied.

#### 3.6.4. Phosphate Solubilization

Phosphate solubilization was observed in 80.65% of bacterial strains, with highly significant differences between strains (one-way ANOVA: F = 116; df = 30 and 62; *p* < 2 × 10^−16^). It is found in all three source compartments, with the highest frequency in soil isolates (100%), followed by cladodes (83.33%) and roots (63.64%). The highest PSI values were recorded for *B. subtilis* R3 (3.41), *P. flexa* S6 (3.39), and *B. subtilis* R6 (3.21) (Figure 5 and Table 10). Overall, phosphate solubilization is a widely distributed trait in this bacterial collection, particularly well represented among soil strains.

#### 3.6.5. Exopolysaccharide Production

Exopolysaccharide production is observed in 35.48% of bacterial strains. It is found in all three source compartments, with a higher frequency in root (45.45%) and cladode (41.67%) strains, while it remains limited in soil strains (12.5%). The highest expression levels are observed in *P. flexa* C6, *B. subtilis* C7, *B. amyloliquefaciens* C8 (Figure 5), *B. amyloliquefaciens* R4, *B. subtilis* R6, and *B. subtilis* S7 (Figure 5 and Table 10). Overall, exopolysaccharide production appears to be a less widely distributed trait.

#### 3.6.6. Amylolytic Activity

Amylolytic activity, present in 35.48% of bacterial strains, remains a secondary trait in the collection. Although it is present in all three compartments studied, its distribution remains heterogeneous and does not suggest any ecological affinity. The highest values are recorded among the *Priestia* (Figure 5 and Table 10).

#### 3.6.7. Lipolytic Activity

Lipolytic activity is observed in 32.26% of bacterial strains, with highly significant differences between strains (one-way ANOVA: F = 152.3; df = 30 and 62; *p* < 2 × 10^−16^). It is present in all three source compartments. A higher frequency is noted among root strains (45.45%) than among cladode (25%) and soil (25%) strains. The highest values are recorded for *B. subtilis* R3 (26.55 mm), *B. subtilis* S7 (23.62 mm), and *B. subtilis* R6 (22.52 mm) (Figure 5 and Table 10). Overall, lipolytic activity appears to be a trait that is weakly represented in this bacterial collection but is more strongly expressed in root isolates.

#### 3.6.8. Proteolytic Activity

Proteolytic activity was detected in 70.97% of the bacterial strains, with highly significant differences between strains (one-way ANOVA: F = 99.08; df = 30 and 62; *p* < 2 × 10^−16^). It is present in all three source compartments, with a frequency of 75% in cladode, and soil isolates, compared to 63.64% in root isolates. The highest values were recorded for *B. amyloliquefaciens* R4 (23.86 mm), *B. tropicus* C12 (18.28 mm) and *B. subtilis* R3 (17.52 mm) (Figure 5 and Table 10). Overall, proteolytic activity appears to be a widely distributed trait in this bacterial collection.

#### 3.6.9. Structuring and Classification of Bacterial Strains Based on Their PGP Traits

**Figure 6:**
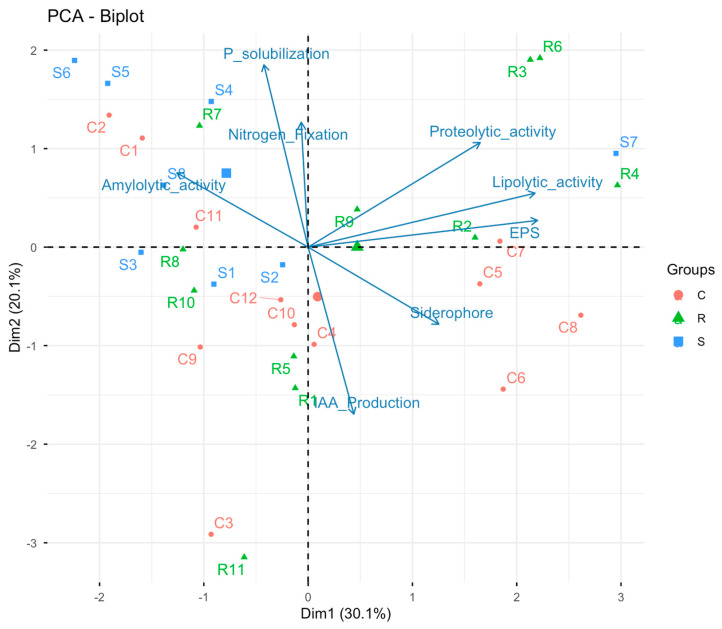
Functional distribution of bacterial strains according to their PGP properties.

The principal component analysis biplot reveals a clear clustering of strains based on their PGP traits (Figure 6). The first two axes together account for 50.2% of the variability, which already allows for the visualization of robust trends. The dimdesc analysis revealed statistically significant associations between variables and the main axes (*p* < 0.05). Axis 1 was primarily correlated with exopolysaccharide (EPS) production as well as lipolytic and proteolytic activities, whereas Axis 2 was associated with phosphorus solubilization, nitrogen fixation, and IAA production. The root isolates, notably R3 (*B. subtilis*), R4 (*B. amyloliquefaciens*), R6 (*B. subtilis*), and R7 (*P. flexa*), cluster in the quadrant associated with high levels of phosphate solubilization, atmospheric nitrogen fixation, and enzymatic activities (protease and lipase), revealing a particularly comprehensive PGP profile. Conversely, several cladode strains such as C3 (*P. rettgeri*), C6 (*P. flexa*), C8 (*B. amyloliquefaciens*), C9 (*S. hominis*), and C11 (*M. yunnanensis*) are positioned in areas strongly correlated with IAA production and siderophores, indicating a more pronounced hormonal or competitive specialization. Soil isolates, including S5, S6, S4, and S8 (all *P. flexa*), are located near the vectors for phosphate solubilization and amylolytic activity, whereas S7 (*B. subtilis*) stands out for its increased enzymatic activities. Overall, this profile highlights a clear specialization based on the compartment of origin: root strains exhibit the most comprehensive PGP traits, edaphic strains are characterized by marked nutrient acquisition capabilities, and cladode strains predominantly include the best auxin producers, revealing strong functional heterogeneity within the microbiota associated with *O. dillenii*.

**Figure 7:**
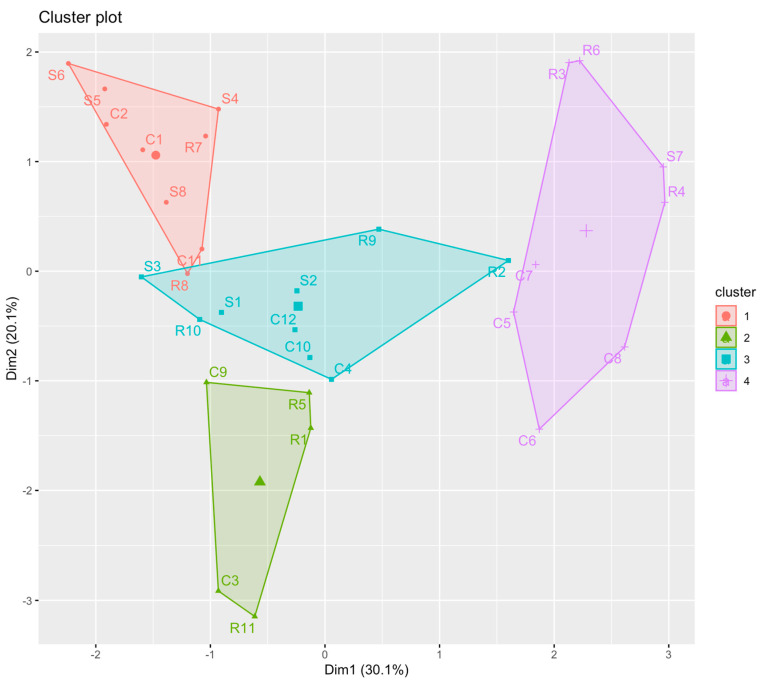
Hierarchical classification of strains based on their PGP traits.

The cluster plot (Figure 7) effectively complements the PCA by organizing the 31 strains into four distinct functional groups. Cluster 1, consisting of C1 (*P. flexa*), C2 (*P. flexa*), C11 (*M. yunnanensis*), S4 (*P. flexa*), S5 (*P. flexa*), S6 (*P. flexa*), S8 (*P. flexa*), R7 (*P. flexa*), and R8 (*B. cereus*), comprises isolates with low to moderate PGP profiles, without marked specialization. Cluster 2, consisting of C3 (*P. rettgeri*), C9 (*S. hominis*), and R11 (*P. rettgeri*), corresponds to the hormonal cluster, as these three strains produce the highest levels of IAA in the collection; strains R1 (*C. sakazakii*) and R5 (*C. sakazakii*), although lacking IAA, are associated with this cluster due to the overall similarity of their functional signatures. Cluster 3, comprising S1 (*M. aborescens*), S2 (*H. oleronia*), S3 (*A. faecalis*), R9 (*B. cereus*), R10 (*P. asaccharolyticum*), as well as C4 (*B. paranthracis*), C10 (*P. flexa*), and C12 (*B. tropicus*), groups strains with intermediate and less specialized profiles, consistent with their central position in the PCA. Finally, cluster 4 groups strains exhibiting the most versatile functional profiles and the highest levels of enzymatic activity: R3 and R6 (*B. subtilis*), R4 (*B. amyloliquefaciens*), S7 (*B. subtilis*), C5, C6, C7 (*B. subtilis*), and C8 (*B. amyloliquefaciens*), characterized by particularly high levels of protease, lipase, and EPS production and, for several, strong phosphate solubilization. Thus, the *cluster plot* highlights a clear functional structure of the *Opuntia dillenii* microbiota, revealing four clusters: a high-performance enzymatic group, a specialized hormonal group, a non-specialized intermediate group, and a low-functional group, complementing and refining the information provided by PCA.

### 3.7. Resilience of Bacterial Strains to pH and Temperature Variations

#### 3.7.1. Resilience of Bacterial Strains to pH Variations

**Figure 8:**
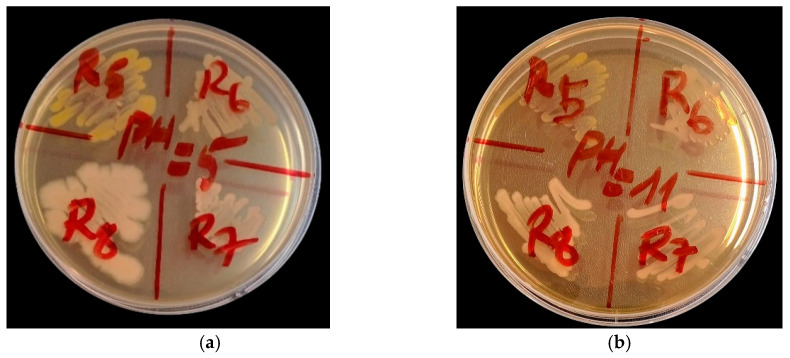
Growth of bacterial strains on NA medium at (**a**) pH 5 and (**b**) 11.

According to the data analysis, the survival of bacterial strains is influenced by the pH of the medium. Optimal tolerance is observed at pH 6, 7, and 8, where all strains survive (100%). Survival remains high at pH 9 and 10 (96.77%), then decreases slightly at pH 11 (93.55%). In an acidic medium (pH 5), a marked decrease is recorded, with a survival rate limited to 45.16% (Table 11). The strains identified as the most tolerant are *B. cereus* (R8 and R9) (Figure 8 and Table 11), *H. oleronia* S2, *A. faecalis* S3, *P. flexa* (S4 and R7), *B. subtilis* R6, *P. asaccharolyticum* R10, and *P. rettgeri* R11.

#### 3.7.2. Resilience of Bacterial Strains to Temperature Variations

**Figure 9:**
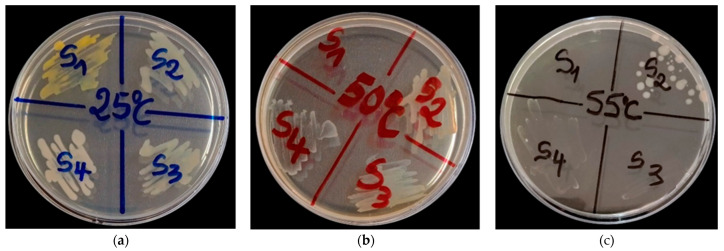
Growth of bacterial strains on NA medium at (**a**) 25 °C, (**b**) 50 °C, and (**c**) 55 °C.

A survival rate of 100% was observed for bacterial strains incubated at temperatures ranging from 25 °C to 40 °C. At 45 °C, survival remains high at 96.77% but drops to 61.29% at 50 °C. At 55 °C, the survival rate falls to 25.81%. The most resilient strains, demonstrating remarkable resilience, are *H. oleronia* S2, *P. flexa* S4 (Figure 9 and Table 11), *B. anthracis* R2, *B. subtilis* R3, *P. flexa* C5, *B. subtilis* C7, and *M. yunnanensis* C11.

## 4. Discussion

In recent years, endophytic bacteria have been extensively reported to colonize internal plant tissues, where they establish mainly beneficial symbiotic associations that enhance plant growth, stress tolerance, and nutrient acquisition [17]. In this study, twenty-three cultivable endophytic bacteria were isolated from *Opuntia dillenii* and successfully identified. Colonization levels were markedly higher in roots than in cladodes, a pattern consistent with numerous studies identifying roots as the primary entry point and preferential niche for endophytic bacteria. Roots are in direct contact with soil, which represents a major microbial reservoir, and may therefore be exposed to a higher influx of microorganisms from the rhizosphere. This close proximity, combined with specific chemical signals, promotes more intense colonization of root tissues compared with aerial organs [57,58,59]. Although aerial tissues can also host endophytes, their colonization is constrained by physical and physiological barriers such as the cuticle, succulence, and exposure to environmental stresses, leading to reduced colonization frequencies. Comparative studies confirm that endophytic colonization is consistently higher in roots than in leaves or other aerial organs across diverse plant species and ecological contexts [60,61]. In addition to endophytes, eight cultivable rhizobacteria were isolated from the rhizosphere of *O. dillenii*, with an average density of 4.3 × 10^5^ CFU·g^−1^. These values are comparable to those reported for the rhizosphere of *Agave potatorum*, an arid-adapted species from Mexico, where bacterial densities of 3.9 × 10^4^ CFU·g^−1^ were observed [62]. Diversity and evenness indices revealed a clear compartment-dependent gradient, with the highest diversity in rhizosphere soil, intermediate levels in cladodes, and the lowest values in roots. This pattern aligns with previous studies demonstrating that soil represents a highly heterogeneous environment offering diverse microhabitats and resources that favor microbial diversity [57,58,59]. Conversely, the root endosphere functions as a highly selective compartment, where host-driven biological and functional filtering leads to dominance by a restricted number of specialized taxa, resulting in reduced diversity and evenness [60,63]. Cladodes occupy an intermediate position; although subjected to strong abiotic constraints such as radiation, desiccation, and temperature fluctuations, they often harbor higher diversity than roots due to less targeted functional selection [61].

The physicochemical properties of the rhizosphere soil further support this ecological framework. The soil exhibited an almost exclusively sandy texture, typical of coastal environments [64,65], which is associated with low cation exchange capacity and limited nutrient retention, as reported for other coastal sandy soils [66,67]. The low salinity observed is consistent with vegetated coastal systems, where precipitation-driven leaching promotes downward salt transport [58]. A pH value of 5.99 places this soil within the sub-acidic range, which is characteristic of leached coastal sandy soils [68]. Numerous studies have demonstrated that soil pH, organic matter content, and macronutrient availability are key drivers of microbial community structure in soils and the rhizosphere [69,70]. In particular, soil pH is widely recognized as a major determinant of bacterial community composition [71], while carbon availability strongly influences the assembly of the rhizosphere microbiome [72]. Together, these findings support a soil–plant continuum model, in which microbial community assembly is governed by the combined selective pressures exerted by soil conditions and host plant filtering. The rhizosphere constitutes the first and most influential level of selection for plant-associated microorganisms. Acting as a powerful ecological filter, it allows the establishment of only those bacteria capable of tolerating the physicochemical stresses typical of rhizosphere soils, including desiccation, salinity, and pH fluctuations [73,74]. From this pre-selected pool, only a limited subset of rhizosphere-adapted bacteria can overcome plant physio-logical barriers and successfully colonize the endosphere, where additional constraints arise from host defense mechanisms [17,75]. This dual filtering process, driven jointly by soil conditions and plant-mediated selection, favors microorganisms endowed with particularly effective survival and adaptation strategies.

The bacterial isolates exhibited marked morphological heterogeneity, reflecting substantial phenotypic diversity within the cultivable community. Most colonies were circular, flat, medium-sized, smooth-surfaced, and opaque, with a predominantly creamy consistency, although dry and mucoid morphotypes were also observed. According to Adeleke and Fakoya [48], colony morphology provides valuable insight into the physiological and genetic characteristics of bacterial strains. Smooth colony surfaces may facilitate adaptation to fluctuating environmental conditions, whereas circular colony architecture has been associated with specific genetic determinants. In addition, a mucoid appearance is commonly linked to exopolysaccharide production, a hallmark of biofilm-forming bacteria. The majority of isolates were Gram-positive and bacillary, a predominance that may be related to carbon-use strategies, as Gram-positive bacteria preferentially exploit recalcitrant carbon sources that are more difficult to degrade, while Gram-negative bacteria rely mainly on readily biodegradable plant-derived compounds [76].

At the taxonomic level, the bacterial community identified in this study was dominated by the phyla Firmicutes, Actinobacteria, and Proteobacteria, a configuration consistently reported in plant-associated microbiomes from arid and semi-arid ecosystems. These environments are characterized by severe abiotic constraints, including limited water availability, pronounced thermal fluctuations, occasional salinity stress, and low nutrient content, all of which exert strong selective pressure on soil microorganisms [17,73,74]. This recurrent taxonomic structure has been documented not only by high-throughput metagenomic approaches but also by culture-dependent studies conducted on desert plants from Africa, the Middle East, and Asia. Despite differences in host plant taxonomy, these studies consistently report a marked convergence in associated bacterial communities dominated by the same major phylogenetic groups [77,78,79]. Among the cultivable bacterial isolates, the community was largely dominated by the genera *Bacillus* and *Priestia*, which accounted for 35.48% and 32.25% of the isolates, respectively. This taxonomic distribution aligns well with previous studies on endophytic bacteria associated with desert and succulent plants. For instance, an investigation of 191 endophytic isolates from the wild cactus *Euphorbia trigona* reported a strong predominance of *Bacillus* (58 isolates), followed by genera such as *Lysinibacillus* and *Pseudomonas* [80]. The ecological success of these taxa is mainly attributed to their capacity to form endospores, which confers resistance to prolonged heat and drought stress, as well as to their remarkable metabolic plasticity [73,75].

Phylogenetic analyses of *Bacillus* isolate underline the well-known taxonomic challenges associated with the *Bacillus cereus* sensu lato complex. In the present study, isolates R8 and R9 clustered within a clade containing *B. cereus* and *B. thuringiensis*, together with closely related taxa such as *B. anthracis* and *B. paranthracis*, without enabling reliable species-level discrimination. These observations confirm that even when markers with higher discriminatory power than the 16S rRNA gene, such as *gyrB*, are employed, species-level resolution within the *B. cereus* complex remains limited due to their high genetic similarity [81,82]. The core members of the complex (*B. cereus*, *B. thuringiensis*, *B. anthracis*) share a very close common ancestor on the evolutionary scale. Their chromosomal backbone has not had enough time to accumulate sufficient neutral mutations through genetic drift to differentiate their 16S rRNA sequences, and numerous DNA-DNA hybridization and genomic studies suggest that they biologically belong to a single species [83,84]. Furthermore, the genome of these bacteria possesses a high number of 16S rRNA gene copies, typically between 11 and 14 operons [85]. To prevent these copies from diverging within the same cell, a repair mechanism called gene conversion permanently corrects nascent mutations by using the other copies as a template, maintaining strict intra-genomic and inter-species homogeneity [3]. Although the *gyrB* gene exhibits a higher mutation rate and better overall resolution than 16S rRNA, its application faces intrinsic taxonomic limitations within the *Bacillus cereus* sensu lato complex. Comparative molecular phylogeny studies [86,87,88] demonstrate that the discriminatory power of *gyrB* remains insufficient to reliably separate *B. cereus* sensu stricto from *B. thuringiensis*, as the sequences of this locus remain highly conserved between these sister taxa. Furthermore, global genomic analyses reveal that the *gyrB* gene undergoes frequent homologous recombination and inter-species horizontal gene transfer (HGT) events within the group [89]. This intense genetic plasticity generates inconsistent phylogenetic trees from one housekeeping gene to another, confirming that an approach based solely on the *gyrB* gene cannot resolve taxonomic uncertainties and necessitates the use of multi-locus sequence analysis (MLSA) or pangenomic approaches. Rutkowska et al. [90] further emphasized that members of this complex frequently contaminate food and raw materials, as their spores display exceptional resistance to dehydration and thermal treatments. From a functional standpoint, *B. cereus* is recognized as an opportunistic pathogen responsible for foodborne illness through the production of the chromosomally encoded toxins Nhe, Hbl, and CytK, as well as the emetic toxin cereulide encoded on the megaplasmid pCER270. In contrast, *B. thuringiensis* is defined by plasmid-encoded *cry*, *cyt*, and *vip* genes that confer insecticidal activity, leading to its widespread use as a biopesticide considered safe for humans. The pathogenicity of *B. anthracis*, in turn, is linked to the plasmids pXO1 and pXO2, which encode the anthrax toxin and the poly-γ-D-glutamate capsule, respectively, illustrating the decisive role of plasmids in functional differentiation within the *B. cereus* complex. Conversely, analysis based on the *gyrB* gene unambiguously confirmed that isolate S6 belongs to *Priestia flexa*. The value of *gyrB* extends beyond its role in DNA supercoiling regulation to its contribution to phylogenetic inference, where its higher mutation rate provides improved resolution for closely related taxa with ≥99% sequence identity [81].

Beyond their abundance, plant-associated *Bacillus* species are well known for their broad functional potential. They are capable of producing lipopeptides, hydrolytic enzymes, siderophores, and a wide array of antimicrobial compounds, while also modulating plant physiological responses to both abiotic and biotic stresses [91,92]. These functional attributes underpin their increasing interest as candidates for biocontrol and plant growth-promotion strategies in sustainable agriculture [93]. Our results have shown that *Bacillus* species, particularly *B. subtilis* and *B. amyloliquefaciens*, exhibit marked expression of plant growth-promoting (PGP) traits. Phosphate solubilization is highly expressed in *B. subtilis* R3 and R6, confirming their ability to mobilize insoluble phosphorus and improve plant phosphorus nutrition, in line with the observations of Iqbal et al. [94] and Fu et al. [95] for *B. subtilis* strains derived from stressed environments. The biological fixation of atmospheric nitrogen, observed in *B. subtilis* C7 and R3, confirms the potential role of these isolates in indirectly improving nitrogen nutrition in plants, as previously reported for free-living diazotrophs associated with plant roots and internal tissues [96,97]. Siderophore production is widespread among *Bacillus* isolates, with particularly high levels in *B. subtilis* S7 and C7. This ability is consistent with the high siderophore potential described for the genus *Bacillus*, which promotes iron uptake by the plant and microbial competition against plant pathogens [98,99]. Several isolates also exhibit significant production of exopolysaccharides (EPS), notably *B. subtilis* (C7, S7, R6) and *B. amyloliquefaciens* (C8, R4). EPS production is associated with adhesion, biofilm formation and improved water retention in the rhizosphere, as demonstrated in *B. subtilis* and *B. amyloliquefaciens* [100,101,102]. The extracellular enzymatic activities observed reinforce the biocontrol potential of *Bacillus* species. Protease production is the most frequent and intense activity, whilst lipase activities vary depending on the isolate. These enzymes are directly involved in the degradation of the cellular structures of fungal and bacterial plant pathogens and constitute a key mechanism of *Bacillus*-mediated biocontrol [103,104]. Taken together, these traits suggest that *Bacillus* isolates may represent promising candidates for plant growth-promotion and biocontrol, as reported in previous studies. These results are consistent with the work of Ling et al. [105], which demonstrates the efficacy of *Bacillus* strains against a broad spectrum of plant pathogens. Finally, the pH and temperature tolerance profiles highlight the high ecological plasticity of *Bacillus* isolates, particularly *B. subtilis* and *B. cereus*. This ability to adapt to multiple stresses (temperature, pH) is attributed to the expression of heat shock proteins, effective metabolic regulation and spore formation, conferring a major adaptive advantage in environments subject to severe abiotic stresses [106,107].

*P. flexa* isolates exhibit marked traits promoting plant growth, notably effective phosphate solubilization (isolate S6) and nitrogen-fixing capacity (isolates S4, S5 and S6), confirming their potential for improving plant mineral nutrition [87]. The production of indole-3-acetic acid (IAA) is also observed, at moderate to high levels depending on the isolate, suggesting a role in stimulating root development and the physiological regulation of the host plant. Enzymatic profiles reveal pronounced amylolytic activity, contrasting with more moderate expression of proteases and lipases, indicating a functional specialization geared towards the degradation of complex polysaccharides rather than direct biocontrol mechanisms [108]. Furthermore, the production of exopolysaccharides by certain isolates (notably C6), combined with good tolerance to variations in pH and temperature, suggests high metabolic stability compatible with the severe environmental constraints characteristic of *O. dillenii* habitats [109]. These results suggest that *P. flexa* may contribute to the functional resilience of the microbiome prioritizing the optimization of plant nutrition and physiology under arid conditions rather than strategies of direct antimicrobial competition.

Despite the strong dominance of *Firmicutes*, the detection of less abundant taxa such as *P. rettgeri*, *C. sakazakii*, *M. arborescens*, *M. yunnanensis*, *A. faecalis*, and *P. asaccharolyticum* indicates substantial functional diversity within the microbiome associated with *O. dillenii*. Recent evidence suggests that these subdominant or “rare” taxa play an important role in maintaining the resilience and functional stability of plant microbiomes under severe environmental stress [77,78,110]. Among the Proteobacteria, *P. rettgeri* and *C. sakazakii* stand out for their ability to survive under harsh environmental conditions, particularly in terms of pH and temperature. In particular, *P. rettgeri* exhibits high production of indole-3-acetic acid (IAA) as well as high production of siderophores, suggesting an involvement in the modulation of the plant’s physiological responses and in competition for iron [111,112]. *C. sakazakii* exhibits several traits associated with plant growth-promoting bacteria, notably the production of EPS and siderophores, contributing to abiotic stress tolerance and improved iron availability. EPS production, linked to biofilm formation, promotes survival under conditions of low water activity and is associated with the activation of betA/betB genes involved in betaine biosynthesis [113]. However, the opportunistic nature of *P. rettgeri* reported in the literature warrants a cautious interpretation of its application potential [114].

Conversely, isolates of *A. faecalis* and *P. asaccharolyticum* exhibit profiles that are less oriented towards classic PGP traits, but more towards functions linked to soil biogeochemical processes, notably tolerance to alkaline pH and persistence under variable physico-chemical conditions. These observations are consistent with the ability of *A. faecalis* to participate in nitrogen transformation and mobilization, notably via heterotrophic nitrification and ammonium oxidation mechanisms such as the dirammox pathway [115], as well as with the role of *P. asaccharolyticum* as a rhizosphere bacterium involved in the indirect improvement of plant nutrition and physiological responses [116].

Finally, the Actinobacteria identified in this study exhibit complementary functional strengths. *M. yunnanensis* is distinguished by good tolerance to abiotic stress, associated with EPS production, high nitrogenase activity and high IAA production, suggesting an indirect role in microbiome resilience and the physiological stimulation of plants. *Microbacterium arborescens*, although lacking classic PGP traits, exhibits IAA production as well as enzymatic activities (proteolytic and lipolytic) involved in the degradation of organic matter, contributing to nutrient recycling and the biogeochemical functioning of the soil. These results are consistent with the roles described for these taxa in the production of secondary metabolites, antioxidant compounds and extracellular enzymes involved in pathogen suppression and the alleviation of oxidative stress, thereby contributing to the overall tolerance of the microbiome associated with *O. dillenii* [117,118].

The occurrence of opportunistic pathogens in soil and plant-associated microbiomes is well documented and can be attributed to various environmental and anthropogenic factors. Livestock activities represent a major source of environmental contamination, as many of these bacteria are commensal inhabitants of the gastrointestinal tract of animals. Grazing or animal husbandry in proximity to agricultural areas may lead to fecal contamination of soils, thereby introducing enteric and opportunistic bacteria into the rhizosphere. Similarly, inadequate sanitation infrastructure may result in the release of human-associated bacteria into the environment, further contributing to the complexity of soil microbial communities. These contexts explain the coexistence of beneficial, neutral, and potentially harmful bacteria within the same soil–plant system. Although some of these taxa have been reported to indirectly contribute to soil functioning or plant performance under certain conditions, their potential use in agriculture requires particular caution [48]. Consequently, the identification of isolates closely related to opportunistic and nosocomial pathogens—specifically *Cronobacter sakazakii*, *Providencia rettgeri*, *Staphylococcus hominis*, and members of the *Bacillus cereus* complex—raises critical biosafety and biosecurity concerns. Utilizing such strains as live agricultural inoculants introduces an unacceptable risk of environmental dissemination and human exposure. Therefore, any future consideration of their practical or commercial application is strictly conditional upon rigorous biosafety assessments, including comprehensive whole-genome pathogenicity profiling, determination of their virulome, and extensive antimicrobial resistance (AMR) phenotyping to rule out multidrug-resistant traits.

Although a composite sampling strategy based on three healthy plants ensured local representativeness of the studied site, the limited number of sampled individuals and the lack of spatial and temporal replication restrict the generalizability of the results. It should also be noted that this study relies exclusively on culture-dependent approaches, which capture only a limited fraction of the total bacterial diversity. Future studies combining culture-independent approaches, such as 16S rRNA gene metabarcoding, would provide a more comprehensive overview of the bacterial communities associated with *Opuntia dillenii* in coastal environments. Furthermore, complementary investigations focusing on well as the assessment of their antibiotic resistance profiles, will be necessary to better evaluate their ecological potential, possible applications, and the associated biosafety considerations.

## 5. Conclusions

This study highlights the potential of microbial resources from coastal areas as a lever for the development of sustainable agricultural approaches suited to constrained environments. It makes a significant contribution to our understanding of cultivable endophytic and rhizosphere bacterial communities associated with *Opuntia dillenii* in the coastal zone of Benin, an environment that remains largely unexplored from a microbiological perspective. The isolated communities were mainly composed of representatives of the phyla Firmicutes, Actinobacteria and Proteobacteria, with a marked dominance of the genera *Bacillus* and *Priestia*. Among the identified species, *P. flexa* emerged as the most frequently isolated species across all the compartments studied, highlighting its remarkable adaptation and central role within the cultivable microbiota associated with *O. dillenii* in this sandy, nutrient-poor coastal habitat. Beyond taxonomic characterization, functional analysis of the isolated strains revealed a wide diversity of traits linked to the promotion of plant growth. These traits include mechanisms involved in nutrient acquisition, phytohormone production, siderophore synthesis, exopolysaccharide production, phosphorus solubilization, and various enzymatic activities. Several strains stood out for their particularly effective and versatile functional profiles, notably S7 (*B. subtilis*), R3 (*B. subtilis*), R6 (*B. subtilis*), R4 (*B. amyloliquefaciens*), C5 (*P. flexa*), C6 (*P. flexa*), C7 (*B. subtilis*) and C8 (*B. amyloliquefaciens*). Despite the clear agronomic potential of several isolates for agricultural systems under stress, the biosafety risks associated with certain strains in the collection necessitate a thorough assessment before their use as bio-inoculants can be considered.

## Figures and Tables

**Table 1:** PCR conditions for amplification of the 16S rDNA gene.

Primers	Steps	Temperatures	Time	Cycles
27F/1492R	Initial denaturation	95 °C	1 min	-
	Denaturation	95 °C	15 s	30 cycles
	Hybridization	56 °C	12 s	
	Extension	72 °C	12 s	
	Final extension	72 °C	20 min	-
	End of PCR	4 °C	∞	-

**Table 2:** PCR conditions for amplification of the 16S *gyrB* gene.

Primers	Steps	Temperatures	Time	Cycles
UP1/UP2R	Initial denaturation	95 °C	5 min	-
	Denaturation	95 °C	30 s	35 cycles
	Hybridization	56.5 °C	30 s	
	Extension	72 °C	1 min 30 s	
	Final extension	72 °C	10 min	-
	End of PCR	4 °C	∞	-

**Table 3:** Physicochemical composition of the rhizosphere soil.

Category	Parameters	Mean ± Standard Deviation (*n* = 3)
Texture	% Clay	0.19 ± 0.03
	% Fine silt	0.00 ± 0.00
	% Coarse silt	0.35 ± 0.05
	% Fine sand	4.93 ± 0.17
	% Coarse sand	93.85 ± 0.31
Organic properties	C (%)	0.29 ± 0.02
	N (%)	0.048 ± 0.005
	C/N ratio	7.00 ± 0.24
	MO (%)	0.50 ± 0.03
Chemical properties	pH	5.99 ± 0.04
	Exchangeable Ca^2+^ (meq/100 g)	1.15 ± 0.06
	Exchangeable Mg^2+^ (meq/100 g)	0.125 ± 0.01
	Exchangeable K^+^ (meq/100 g)	0.383 ± 0.02
	Exchangeable Na^+^ (meq/100 g)	0.326 ± 0.02
	Sum of exchangeable cations (meq/100 g)	1.989 ± 0.08
	CEC (meq/100 g)	2.213 ± 0.13
	TDS (%)	89.88 ± 0.75
	Assimilable P (mg/kg)	6.00 ± 0.28
Soil solution	TDS (mg/L)	248 ± 3.3
	Salinity (psu)	0.17 ± 0.02
	EC (µS/cm)	281 ± 12

**Table 4:** Macroscopic and microscopic aspects of endophytic bacterial isolates (Cladodes).

Isolate Code	Shape	Elevation	Size	Surface	Opacity	Color	Consistency	Margin	Gram	Shape	Arrangement
C1	Circular	Flat	Medium	Smooth	Opaque	Whitish	Creamy	Entire	G+	Rod	Single
C2	Circular	Flat	Medium	Smooth	Opaque	Whitish	Creamy	Entire	G+	Rod	Pairs
C3	Circular	Convex	Small	Smooth	Translucent	Whitish	Creamy	Entire	G−	Rod	Single
C4	Irregular	Flat	Large	Rough	Opaque	Whitish	Dry	Wavy	G+	Rod	Single
C5	Circular	Flat	Medium	Smooth	Opaque	Whitish	Creamy	Entire	G+	Rod	Pairs
C6	Circular	Flat	Medium	Smooth	Opaque	Whitish	Creamy	Entire	G+	Rod	Pairs
C7	Circular	Flat	Medium	Rough	Opaque	Whitish	Dry	Wavy	G+	Rod	Pairs
C8	Irregular	Flat	Medium	Rough	Opaque	Whitish	Mucoid	Wavy	G+	Rod	Pairs
C9	Circular	Convex	Small	Smooth	Opaque	Whitish	Creamy	Entire	G+	Cocci	Staphylococci
C10	Circular	Flat	Medium	Smooth	Opaque	Whitish	Creamy	Entire	G+	Rod	Pairs
C11	Circular	Convex	Punctiform	Smooth	Opaque	Bright yellow	Creamy	Entire	G+	Cocci	Tetrad
C12	Irregular	Flat	Large	Rough	Opaque	Whitish	Dry	Wavy	G+	Rod	Pairs

**Table 5:** Macroscopic and microscopic aspects of endophytic bacterial isolates (Roots).

Isolate Code	Shape	Elevation	Size	Surface	Opacity	Color	Consistency	Margin	Gram	Shape	Arrangement
R1	Circular	Convex	Medium	Smooth	Translucent	Yellow	Creamy	Entire	G-	Rod	Pairs
R2	Irregular	Flat	Large	Rough	Opaque	Whitish	Dry	Wavy	G+	Rod	Single
R3	Circular	Flat	Medium	Rough	Opaque	Whitish	Dry	Wavy	G+	Rod	Pairs
R4	Irregular	Flat	Medium	Rough	Opaque	Whitish	Mucoid	Wavy	G+	Rod	Pairs
R5	Circular	Convex	Medium	Smooth	Translucent	Yellow	Creamy	Entire	G-	Rod	Pairs
R6	Circular	Flat	Medium	Rough	Opaque	Whitish	Dry	Wavy	G+	Rod	Pairs
R7	Circular	Flat	Medium	Smooth	Opaque	Whitish	Creamy	Entire	G+	Rod	Pairs
R8	Irregular	Flat	Large	Rough	Opaque	Whitish	Dry	Wavy	G+	Rod	Single
R9	Irregular	Flat	Large	Rough	Opaque	Whitish	Dry	Wavy	G+	Rod	Single
R10	Circular	Convex	Small	Smooth	Opaque	Yellowish	Creamy	Entire	G-	Rod	Single
R11	Circular	Convex	Small	Smooth	Translucent	Whitish	Creamy	Entire	G-	Rod	Single

**Table 6:** Macroscopic and microscopic aspects of endophytic bacterial isolates (Soil).

Isolate Code	Shape	Elevation	Size	Surface	Opacity	Color	Consistency	Margin	Gram	Shape	Arrangement
S1	Circular	Convex	Small	Smooth	Opaque	Yellow	Creamy	Entire	G+	Rod	Single
S2	Circular	Convex	Small	Smooth	Translucent	Whitish	Creamy	Entire	G+	Rod	Single
S3	Circular	Convex	Small	Smooth	Translucent	Whitish	Creamy	Entire	G-	Rod	Single
S4	Circular	Flat	Medium	Smooth	Opaque	Whitish	Creamy	Entire	G+	Rod	Pairs
S5	Circular	Flat	Medium	Smooth	Opaque	Whitish	Creamy	Entire	G+	Rod	Pairs
S6	Circular	Flat	Medium	Smooth	Opaque	Whitish	Creamy	Entire	G+	Rod	Pairs
S7	Circular	Flat	Medium	Rough	Opaque	Whitish	Dry	Wavy	G+	Rod	Pairs
S8	Circular	Flat	Medium	Smooth	Opaque	Whitish	Creamy	Entire	G+	Rod	Pairs

**Table 7:** NCBI BLAST 16S rRNA gene sequences of bacterial endophytes isolated.

Origin/Source	Codes	GenBank Accession No.	NCBI Blast Results	% Similarity
Cladode	C1	PZ102195	*Priestia flexa* KT719725.1	100%
	C2	PZ102196	*Priestia flexa* PQ168997.1	100%
	C3	PZ102197	*Providencia rettgeri* MZ895029.1	99.87%
	C4	PZ102198	*Bacillus anthracis* PQ461949.1	100%
	C5	PZ102199	*Priestia flexa* OR814211.1	100%
	C6	PZ102200	*Priestia flexa* OR514189.1	100%
	C7	PZ102201	*Bacillus subtilis* PQ780361.1	99.75%
	C8	PZ102202	*Bacillus amyloliquefaciens* MT826227.1	100%
	C9	PZ102203	*Staphylococcus hominis* MZ712950.1	99.74%
	C10	PZ102204	*Priestia flexa* KT719725.1	100%
	C11	PZ102205	*Micrococcus yunnanensis* MT033093.1	100%
	C12	PZ102206	*Bacillus cereus* AP022907.1	99.91%
Root	R1	PZ102207	*Cronobacter sakazakii* HQ880343.1	99.47%
	R2	PZ102208	*Bacillus anthracis* MW819988.1	100%
	R3	PZ102209	*Bacillus subtilis* MK103123.1	100%
	R4	PZ102210	*Bacillus amyloliquefaciens* MT826227.1	100%
	R5	PZ102211	*Cronobacter sakazakii* JQ963900.1	100%
	R6	PZ102212	*Bacillus subtilis* MK103123.1	100%
	R7	PZ102213	*Priestia flexa* KT758531.1	100%
	R8	PZ102214	*Bacillus cereus* KF494193.1	99.68%
	R9	PZ102215	*Bacillus cereus* KU922281.1	100%
	R10	PZ102216	*Pseudochrobactrum asaccharolyticum* PX481839.1	100%
	R11	PZ102217	*Providencia rettgeri* PP980523.1	100%
Soil	S1	PZ102218	*Microbacterium aborescens* PQ578210.1	100%
	S2	PZ102219	*Heyndrickxia oleronia* MF662514.1	100%
	S3	PZ102220	*Alcaligenes faecalis* ON028636.1	100%
	S4	PZ102221	*Priestia flexa* KT719725.1	100%
	S5	PZ102222	*Priestia flexa* PP863229.1	100%
	S6	PZ102223	*Priestia flexa* PX285880.1 *Priestia megaterium* OM304331.1	100%
	S7	PZ102224	*Bacillus subtilis* subsp.*Subtilis* PV168344.1	100%
	S8	PZ102225	*Priestia flexa* KT719725.1	100%

**Table 8:** α-diversity indices.

Compartment	S	Shannon (H′)	Simpson_1-D	Equity (J′)
Cladodes	5	1.35	0.69	0.84
Roots	5	1.29	0.64	0.80
Soil	5	1.39	0.69	0.86
Overall	10	1.75	0.76	0.76

**Table 9:** NCBI BLAST *gyrB* sequences of bacterial isolates.

Origin/Source	Codes	GenBank Accession No.	NCBI Blast Results	% Similarity
Cladode	C1	PZ149653	*Priestia flexa* CP120590.2	99.89%
	C2	PZ149654	*Priestia flexa* CP120590.2	99.61%
	C4	PZ149655	*Bacillus paranthracis* CP101135.1	100%
	C5	PZ149656	*Priestia flexa* CP120590.2	99.89%
	C6	PZ149657	*Priestia flexa* CP120590.2	99.29%
	C7	PZ149658	*Bacillus subtilis* CP174496.1	100%
	C8	PZ149659	*Bacillus amyloliquefaciens* CP195015.1	99.89%
	C10	PZ149660	*Priestia flexa* CP120590.2	99.86%
	C12	PZ149661	*Bacillus tropicus* CP053955.1	99.82%
Root	R2	PZ149662	*Bacillus anthracis* CP126515.1	99.34%
	R3	PZ149663	*Bacillus subtilis* CP174496.1	99.89%
	R4	PZ149664	*Bacillus amyloliquefaciens* CP195015.1	100%
	R6	PZ149665	*Bacillus subtilis* CP174496.1	99.78%
	R7	PZ149666	*Priestia flexa* CP120590.2	99.89%
	R8	PZ149667	*Bacillus thuringiensis* CP010088.1 *Bacillus cereus* KF022228.1	100%
	R9	PZ149668	*Bacillus thuringiensis* CP010088.1 *Bacillus cereus* KF022228.1	100%
Soil	S4	PZ149669	*Priestia flexa* CP120590.2	99.55%
	S5	PZ149670	*Priestia flexa* CP120590.2	99.87%
	S6	PZ149671	*Priestia flexa* CP120590.2	99.44%
	S7	PZ149672	*Bacillus subtilis* CP017112.1	100%
	S8	PZ149673	*Priestia flexa* CP120590.2	99.31%

**Table 10:** PGP traits of bacterial strains.

Origin/Sources	Codes	Identity	EPS	Nitrogenase	Amylase	Siderophore $\bar{x}\pm \sigma_{\bar{x}}$ (mm)	Phosphatase (PSI)	IAA (µg/mL)	Protease $\bar{x}\pm \sigma_{\bar{x}}$ (mm)	Lipase $\bar{x}\pm \sigma_{\bar{x}}$ (mm)
Cladode	C1	*P. flexa*	-	++	+	0.00 ± 0.00 ^k^	3.05 ± 0.01 ^abc^	0.00 ± 0.00 ^k^	8.65 ± 0.11 ^ef^	0.00 ± 0.00 ^f^
	C2	*P. flexa*	-	++	++	0.00 ± 0.00 ^k^	2.78 ± 0.10 ^bcde^	0.00 ± 0.00 ^k^	7.52 ± 0.46 ^ef^	0.00 ± 0.00 ^f^
	C3	*P. rettgeri*	-	-	++	30.37 ± 0.8 ^a^	0.00 ± 0.00 ^j^	90.94 ± 1.92 ^c^	0.00 ± 0.00 ^g^	0.00 ± 0.00 ^f^
	C4	*B. paranthracis*	-	-	-	26.50 ± 1.32 ^abc^	1.93 ± 0.06 ^g^	35.52 ± 1.51 ^j^	13.64 ± 0.12 ^d^	0.00 ± 0.00 ^f^
	C5	*P. flexa*	++	+	-	0.00 ± 0.00 ^k^	1.89 ± 0.06 ^g^	68.08 ± 1.43 ^e^	13.99 ± 1.00 ^d^	16.18 ± 0.06 ^cd^
	C6	*P. flexa*	+++	+	-	0.00 ± 0.00 ^k^	0.00 ± 0.00 ^j^	71.94 ± 1.03 ^d^	9.14 ± 0.18 ^ef^	13.91 ± 1.62 ^de^
	C7	*B.* *subtilis*	+++	+++	-	27.96 ± 3.07 ^ab^	1.48 ± 0.10 ^hi^	47.34 ± 1.06 ^h^	15.03 ± 0.77 ^cd^	0.00 ± 0.00 ^f^
	C8	*B. amyloliquefaciens*	+++	+	-	16.62 ± 0.00 ^efg^	1.49 ± 0.00 ^hi^	62.34 ± 0.81 ^g^	14.62 ± 0.01 ^cd^	17.48 ± 1.68 ^c^
	C9	*S. hominis*	-	+++	-	8.325 ± 0.00 ^j^	1.56 ± 0.10 ^ghi^	172.88 ± 0.11 ^a^	0.00 ± 0.00 ^g^	0.00 ± 0.00 ^f^
	C10	*Priestia flexa*	-	+	-	13.29 ± 0.04 ^ghi^	2.51 ± 0.01 ^def^	65.14 ± 0.68 ^f^	9.51 ± 0.19 ^e^	0.00 ± 0.00 ^f^
	C11	*M. yunnanensis*	+	+++	+	8.32 ± 0.01 ^j^	2.38 ± 0.02 ^def^	47.79 ± 1.88 ^h^	0.00 ± 0.00 ^g^	0.00 ± 0.00 ^f^
	C12	*B. tropicus*	-	-	-	0.00 ± 0.00 ^k^	1.61 ± 0.00 ^g^	40.58 ± 1.14 ^i^	18.28 ± 0.87 ^b^	0.00 ± 0.00 ^f^
Root	R1	*C. sakazakii*	+	++	-	20.96 ± 1.44 ^days^	0.00 ± 0.00 ^j^	0.00 ± 0.00 ^k^	0.00 ± 0.00 ^g^	0.00 ± 0.00 ^f^
	R2	*B. anthracis*	-	-	-	26.45 ± 2.68 ^abc^	1.74 ± 0.00 ^gh^	0.00 ± 0.00 ^k^	16.23 ± 2.21 ^bcd^	21.61 ± 0.64 ^b^
	R3	*B.* *subtilis*	++	+++	-	15.73 ± 0.67 ^fgh^	3.41 ± 0.12 a	0.00 ± 0.00 ^k^	17.52 ± 0.01 ^bc^	26.55 ± 2.09 ^a^
	R4	*B. amyloliquefaciens*	+++	+	-	24.74 ± 0.48 ^bc^	1.28 ± 0.00 i	0.00 ± 0.00 ^k^	23.86 ± 1.56 ^a^	21.10 ± 2.02 ^b^
	R5	*C. sakazakii*	+	+++	-	20.08 ± 0.02 ^def^	0.00 ± 0.00 ^j^	0.00 ± 0.00 ^k^	0.00 ± 0.00 ^g^	0.00 ± 0.00 ^f^
	R6	*B.* *subtilis*	+++	++	++	22.99 ± 0.84 ^cd^	3.21 ± 0.07 ^ab^	0.00 ± 0.00 ^k^	16.02 ± 0.67 ^bcd^	22.52 ± 0.95 ^b^
	R7	*Priestia flexa*	-	++	++	18.7 ± 0.23 ^ef^	2.68 ± 0.15 ^cdef^	0.00 ± 0.00 ^k^	9.98 ± 0.80 ^e^	0.00 ± 0.00 ^f^
	R8	*Bacillus cereus*	-	-	+++	0.00 ± 0.00 ^k^	0.00 ± 0.00 ^j^	0.00 ± 0.00 ^k^	15.34 ± 0.43 ^cd^	0.00 ± 0.00 ^f^
	R9	*Bacillus cereus*	-	-	-	12.23 ± 0.03 ^hij^	2.28 ± 0.00 ^f^	0.00 ± 0.00 ^k^	13.39 ± 0.80 ^d^	14.63 ± 0.69 ^de^
	R10	*P. asaccharolyticum*	-	-	-	17.68 ± 1.12 ^ef^	2.69 ± 0.23 ^cdef^	0.00 ± 0.00 ^k^	0.00 ± 0.00 ^g^	0.00 ± 0.00 ^f^
	R11	*P. rettgeri*	-	-	+	26.92 ± 0.63 ^abc^	0.00 ± 0.00 ^j^	96.22 ± 2.92 ^b^	0.00 ± 0.00 ^g^	0.00 ± 0.00 ^f^
Soil	S1	*M. aborescens*	-	-	-	9.47 ± 0.00 ^ij^	2.84 ± 0.23 ^bcd^	33.05 ± 0.48 ^j^	8.92 ± 1.04 ^ef^	0.00 ± 0.00 ^f^
	S2	*H. oleronia*	-	-	-	11.14 ± 0.48 ^ij^	2.99 ± 0.00 ^abc^	0.00 ± 0.00 ^k^	0.00 ± 0.00 ^g^	12.17 ± 0.90 ^e^
	S3	*A. faecalis*	-	-	-	0.00 ± 0.00 ^k^	2.36 ± 0.02 ^ef^	0.00 ± 0.00 ^k^	0.00 ± 0.00 ^g^	0.00 ± 0.00 ^f^
	S4	*P. flexa*	-	+++	-	0.00 ± 0.00 ^k^	2.74 ± 0.36 ^cdef^	0.00 ± 0.00 ^k^	10.25 ± 1.02 ^e^	0.00 ± 0.00 ^f^
	S5	*P. flexa*	-	+++	++	0.00 ± 0.00 ^k^	3.06 ± 0.08 ^abc^	0.00 ± 0.00 ^k^	8.39 ± 0.82 ^ef^	0.00 ± 0.00 ^f^
	S6	*P. flexa*	-	+++	+++	0.00 ± 0.00 ^k^	3.39 ± 0.05 ^a^	0.00 ± 0.00 ^k^	6.24 ± 0.39 ^f^	0.00 ± 0.00 ^f^
	S7	*B.* *subtilis*	+++	++	-	27.88 ± 3.59 ^ab^	1.28 ± 0.03 ^i^	0.00 ± 0.00 ^k^	16.33 ± 0.54 ^bcd^	23.62 ± 1.20 ^b^
	S8	*P. flexa*	-	+	++	12.72 ± 0.51 ^ghi^	2.41 ± 0.11 ^def^	0.00 ± 0.00 ^k^	8.04 ± 0.11 ^ef^	0.00 ± 0.00 ^f^

Legend: -: no activity, +: low activity, ++: moderate activity, +++: high activity; $\bar{x}$: Mean; $\sigma_{\bar{x}}$: Standard Error; Within the same column, means marked with different letters are significantly different at the 5% level according to the Student-Newman-Keuls test.

**Table 11:** pH and temperature growth ranges of bacterial strains.

Origin/Source	Codes	Identity	Temperature (°C)	pH
Cladode	C1	*P. flexa*	[25; 50]	[6; 11]
	C2	*P. flexa*	[25; 45]	[6; 11]
	C3	*P. rettgeri*	[25; 45]	[6; 11]
	C4	*B. paranthracis*	[25; 45]	[6; 11]
	C5	*P. flexa*	[25; 55]	[6; 11]
	C6	*P. flexa*	[25; 45]	[6; 11]
	C7	*B.* *subtilis*	[25; 55]	[6; 11]
	C8	*B. amyloliquefaciens*	[25; 50]	[6; 11]
	C9	*S. hominis*	[25; 50]	[6; 11]
	C10	*Priestia flexa*	[25; 45]	[6; 11]
	C11	*M. yunnanensis*	[25; 55]	[6; 11]
	C12	*B. tropicus*	[25; 45]	[6; 11]
Root	R1	*C. sakazakii*	[25; 50]	[5; 11]
	R2	*B. anthracis*	[25; 55]	[5; 11]
	R3	*B.* *subtilis*	[25; 55]	[5; 8]
	R4	*B. amyloliquefaciens*	[25; 50]	[5; 11]
	R5	*C. sakazakii*	[25; 45]	[5; 11]
	R6	*B.* *subtilis*	[25; 50]	[5; 11]
	R7	*Priestia flexa*	[25; 50]	[5; 11]
	R8	*Bacillus cereus*	[25; 45]	[5; 11]
	R9	*Bacillus cereus*	[25; 45]	[5; 11]
	R10	*P. asaccharolyticum*	[25; 45]	[5; 11]
	R11	*P. rettgeri*	[25; 55]	[5; 11]
Soil	S1	*M. aborescens*	[25; 40]	[6; 10]
	S2	*H. oleronia*	[25; 55]	[5; 11]
	S3	*A. faecalis*	[25; 50]	[5; 11]
	S4	*P. flexa*	[25; 55]	[5; 11]
	S5	*P. flexa*	[25; 50]	[6; 11]
	S6	*P. flexa*	[25; 50]	[6; 11]
	S7	*B.* *subtilis*	[25; 45]	[6; 11]
	S8	*P. flexa*	[25; 50]	[6; 11]

## Data Availability

All sequences were registered in the NCBI Nucleotide database (https://www.ncbi.nlm.nih.gov/nucleotide/) (accessed on 23 March 2026) under the following Accession Numbers PZ102195-PZ102225 for 16S sequences and PZ149653-PZ149673 for *gyrB* sequences, respectively.

## References

[1] United Nations (2024). World Population Prospects 2024: Summary of Results.

[2] Ajeethan N., Abbey L., Yurgel S.N. (2026). Role of Plant GrowthPromoting Microbes in Plant Growth and Development. Appl. Microbiol..

[3] Rojas-Rojas F.U., Gómez-Vázquez I.M., Estrada-de Los Santos P., Shimada-Beltrán H., Vega-Arreguín J.C. (2025). The Potential of Paraburkholderia Species to Enhance Crop Growth. World J. Microbiol. Biotechnol..

[4] Timofeeva A.M., Galyamova M.R., Sedykh S.E. (2023). Plant Growth-Promoting Soil Bacteria: Nitrogen Fixation, Phosphate Solubilization, Siderophore Production, and Other Biological Activities. Plants.

[5] Maciel-Rodríguez M., Moreno-Valencia F.D., Plascencia-Espinosa M. (2025). The Role of Plant Growth-Promoting Bacteria in Soil Restoration: A Strategy to Promote Agricultural Sustainability. Microorganisms.

[6] Hiltner L. (1904). Über neuere Erfahrungen und Probleme auf dem Gebiet der Bodenbakteriologie unter besonderer Berücksichtigung der Gründüngung und Brache. Arb. Der Dtsch. Landwirtsch. Ges..

[7] Pacome A.N., Nadege A.A., Emma W.G., Hafiz A.S., Farid B.-M., Adolphe A., Simeon O.K., Lamine B.-M. (2015). Metabolic and Biofungicidal Properties of Maize Rhizobacteria for Growth Promotion and Plant Disease Resistance. Afr. J. Biotechnol..

[8] Wang Y., Wang X., Lan W., Wei Y., Xu F., Xu H. (2021). Impacts and Tolerance Responses of *Coprinus comatus* and *Pleurotus cornucopiae* on Cadmium Contaminated Soil. Ecotoxicol. Environ. Saf..

[9] Tran L., Duc C., Pham D., Dai T., Tuan N. (2025). Bioprospecting Endophytic Bacteria in *Curcuma zedoaria* for In Vitro Antioxidant and Anti-Inflammatory Potentials. Trop. J. Nat. Prod. Res..

[10] Khan T., Alanazi A.K., Sohail M. (2025). Enzymatic Saccharification of the Polysaccharides in *Arthrocaulon macrostachyum* Biomass: Characterization and Application in Methylene Blue Removal. Int. J. Biol. Macromol..

[11] Anand U., Pal T., Yadav N., Singh V.K., Tripathi V., Choudhary K.K., Shukla A.K., Sunita K., Kumar A., Bontempi E. (2023). Current Scenario and Future Prospects of Endophytic Microbes: Promising Candidates for Abiotic and Biotic Stress Management for Agricultural and Environmental Sustainability. Microb. Ecol..

[12] Osayande I.S., Han X., Tsuda K. (2025). Dynamic Shifts in Plant-Microbe Relationships. Plant Biotechnol..

[13] Ahmad Z., Wu J., Chen L., Dong W. (2017). Isolated *Bacillus subtilis* Strain 330-2 and Its Antagonistic Genes Identified by the Removing PCR. Sci. Rep..

[14] Andreozzi A., Prieto P., Mercado-Blanco J., Monaco S., Zampieri E., Romano S., Valè G., Defez R., Bianco C. (2019). Efficient Colonization of the Endophytes *Herbaspirillum huttiense* RCA24 and *Enterobacter cloacae* RCA25 Influences the Physiological Parameters of Oryza Sativa L. Cv. Baldo Rice. Environ. Microbiol..

[15] Krause A., Julich H., Mankar M., Reinhold-Hurek B. (2017). The Regulatory Network Controlling Ethanol-Induced Expression of Alcohol Dehydrogenase in the Endophyte *Azoarcus* sp. Strain BH72. Mol. Plant Microbe Interact..

[16] Ludueña L.M., Anzuay M.S., Angelini J.G., McIntosh M., Becker A., Rupp O., Goesmann A., Blom J., Fabra A., Taurian T. (2019). Genome Sequence of the Endophytic Strain *Enterobacter* sp. J49, a Potential Biofertilizer for Peanut and Maize. Genomics.

[17] Mametja N.M., Ramadwa T.E., Managa M., Masebe T.M. (2025). Recent Advances and Developments in Bacterial Endophyte Identification and Application: A 20-Year Landscape Review. Plants.

[18] Passari A.K., Upadhyaya K., Singh G., Abdel-Azeem A.M., Thankappan S., Uthandi S., Hashem A., Abd_Allah E.F., Malik J.A., As A. (2019). Enhancement of Disease Resistance, Growth Potential, and Photosynthesis in Tomato (*Solanum lycopersicum*) by Inoculation with an Endophytic Actinobacterium, *Streptomyces thermocarboxydus* Strain BPSAC147. PLoS ONE.

[19] Saranraj P., Sayyed R.Z., Kokila M., Salomi V., Sivasakthivelan P., Manigandan M., Mawar R., Mawar R., Sayyed R.Z., Sharma S.K., Sattiraju K.S. (2023). Evolving Concepts of Biocontrol of Phytopathogens by Endophytic *Pseudomonas fluorescence*. Plant Growth Promoting Microorganisms of Arid Region.

[20] Tavares M.J., Nascimento F.X., Glick B.R., Rossi M.J. (2018). The Expression of an Exogenous ACC Deaminase by the Endophyte *Serratia grimesii* BXF1 Promotes the Early Nodulation and Growth of Common Bean. Lett. Appl. Microbiol..

[21] Shoukat R., Cappai M., Pia G., Pilia L. (2023). An Updated Review: *Opuntia ficus indica* (OFI) Chemistry and Its Diverse Applications. Appl. Sci..

[22] Miller J.O., De Barros P.R., Schulenburg A.N., Tully K.L. (2025). Coastal Stressors Reduce Crop Yields and Alter Soil Nutrient Dynamics in Low-Elevation Farmlands. Discov. Agric..

[23] Castle S.C., Samac D.A., Sadowsky M.J., Rosen C.J., Gutknecht J.L.M., Kinkel L.L. (2019). Impacts of Sampling Design on Estimates of Microbial Community Diversity and Composition in Agricultural Soils. Microb. Ecol..

[24] Ratnaweera P.B., De Silva E.D., Williams D.E., Andersen R.J. (2015). Antimicrobial Activities of Endophytic Fungi Obtained from the Arid Zone Invasive Plant *Opuntia dillenii* and the Isolation of Equisetin, from Endophytic *Fusarium* sp. BMC Complement Altern. Med..

[25] Marchut-Mikołajczyk O., Chlebicz M., Kawecka M., Michalak A., Prucnal F., Nielipinski M., Filipek J., Jankowska M., Perek Z., Drożdżyński P. (2023). Endophytic Bacteria Isolated from *Urtica dioica* L.—Preliminary Screening for Enzyme and Polyphenols Production. Microb. Cell Fact..

[26] Speck Μ.L. (1976). Compendium of Methods for the Microbiological Examination of Foods.

[27] (2024). Microbiology of the Food Chain—General Requirements and Guidance for Microbiological Examinations.

[28] (2024). Microbiologie de La Chaîne Alimentaire—Exigences Générales et Recommandations Pour Les Examens Microbiologiques.

[29] Fasusi O.A., Amoo A.E., Babalola O.O. (2021). Characterization of Plant Growth-Promoting Rhizobacterial Isolates Associated with Food Plants in South Africa. Antonie Van Leeuwenhoek.

[30] Stokes G.G. On the Effect of the Internal Friction of Fluids on the Motion of Pendulums. Transactions of the Cambridge Philosophical Society, Part II, 9, 8-106. Scientific Research Publishing. https://www.scirp.org/reference/referencespapers?referenceid=1109859.

[31] Aguirre J. (2023). Introduction. The Kjeldahl Method: 140 Years.

[32] Walkley A., Black I.A. (1934). An Examination of The Degtjareff Method for Determining Soil Organic Matter, and A Proposed Modification of The Chromic Acid Titration Method. Soil Sci..

[33] Bray R.H., Kurtz L.T. (1945). Determination of Total, Organic, and Available Forms of Phosphorus in Soils. Soil Sci..

[34] Schollenberger C.J., Simon R.H. (1945). Determination of Exchange Capacity and Exchangeable Bases in Soil—Ammonium Acetate Method. Soil Sci..

[35] (2023). Qualité Du Sol—Détermination de La Capacité d’échange Cationique (CEC) Potentielle et de La Teneur En Cations Échangeables, à l’aide d’une Solution Molaire d’acétate d’ammonium Tamponnée à pH 7.

[36] Oman S.F., Camões M.F., Powell K.J., Rajagopalan R., Spitzer P. (2007). Guidelines for Potentiometric Measurements in Suspensions Part A. The Suspension Effect (IUPAC Technical Report). Pure Appl. Chem..

[37] FAO (2021). GLOSOLAN Standard Operating Procedures (SOPs).

[38] Riegel P., Archambaud M., Clavé D., Vergnaud M. (2006). Bactéries de Culture et D’identification Difficiles.

[39] Ripoll J., Bon M.-C., Jones W. (2011). Optimization of the genomic DNA extraction method of silverleaf nightshade (*Solanum elaeagnifolium* Cav.), an invasive plant in the cultivated areas within the Mediterranean region. Biotechnol. Agron. Société Environ..

[40] Lane D.J. (1991). 16S/23S rRNA Sequencing. Nucleic Acid Techniques in Bacterial Systematics.

[41] Turner S., Pryer K.M., Miao V.P.W., Palmer J.D. (1999). Investigating Deep Phylogenetic Relationships among Cyanobacteria and Plastids by Small Subunit rRNA Sequence Analysis1. J. Eukaryot. Microbiol..

[42] Yamamoto S., Harayama S. (1995). PCR Amplification and Direct Sequencing of gyrB Genes with Universal Primers and Their Application to the Detection and Taxonomic Analysis of Pseudomonas Putida Strains. Appl. Environ. Microbiol..

[43] Altschul S.F., Gish W., Miller W., Myers E.W., Lipman D.J. (1990). Basic Local Alignment Search Tool. J. Mol. Biol..

[44] Gordon S.A., Weber R.P. (1951). Colorimetric Estimation of Indoleacetic Acid. Plant Physiol..

[45] Khamna S., Yokota A., Lumyong S. (2009). Actinomycetes Isolated from Medicinal Plant Rhizosphere Soils: Diversity and Screening of Antifungal Compounds, Indole-3-Acetic Acid and Siderophore Production. World J. Microbiol. Biotechnol..

[46] Schwyn B., Neilands J.B. (1987). Universal Chemical Assay for the Detection and Determination of Siderophores. Anal. Biochem..

[47] Jensen H.L. (1954). THE AZOTOBACTERIACEAE. Bacteriol. Rev..

[48] Adeleke B.S., Fakoya S. (2025). Isolation and Molecular Characterization of Potential Plant Growth-Promoting Bacteria from Groundnut and Maize. Int. J. Plant Biol..

[49] Carvalhais L.C., Dennis P.G. (2021). The Plant Microbiome: Methods and Protocols. Methods in Molecular Biology.

[50] Masi C., Tebiso A., Selva Kumar K.V. (2023). Isolation and Characterization of Potential Multiple Extracellular Enzyme-Producing Bacteria from Waste Dumping Area in Addis Ababa. Heliyon.

[51] Dogan G., Taskin B. (2021). Hydrolytic Enzymes Producing Bacterial Endophytes of Some Poaceae Plants. Pol. J. Microbiol..

[52] Adeleke B.S., Fakoya S. (2025). Isolation, Screening, and Molecular Identification of Plant Growth-Promoting Rhizobacteria from Maize Rhizosphere Soil. Curr. Appl. Sci. Technol..

[53] Sáhó A., Karikás V., Ásványi B., Lakatos E., Varga L., Greff B. (2024). Bioactive Potential of Actinobacteria Strains Isolated from the Rhizosphere of Lavender, Lemon Balm, and Oregano. Agriculture.

[54] Zverev A.O., Kichko A.A., Pinaev A.G., Provorov N.A., Andronov E.E. (2021). Diversity Indices of Plant Communities and Their Rhizosphere Microbiomes: An Attempt to Find the Connection. Microorganisms.

[55] Goretzko D., Bühner M. (2022). Factor Retention Using Machine Learning With Ordinal Data. Appl. Psychol. Meas..

[56] Lê S., Josse J., Husson F. (2008). FactoMineR: An R Package for Multivariate Analysis. J. Stat. Softw..

[57] Wicaksono W.A., Köberl M., White R.A., Jansson J.K., Jansson C., Cernava T., Berg G. (2024). Plant-Specific Microbial Diversity Facilitates Functional Redundancy at the Soil-Root Interface. Plant Soil.

[58] Yang C., Chen H., Feng X., Zheng C., Liu X., Zhu F. (2023). Soil Physicochemical Properties and Salt Leaching Associated with Typical Plant Communities in Coastal Saline Land. J. Soil Sci. Plant Nutr..

[59] Yang H., Zheng Y., Yang Z., Wang Q.-C., Lü P.-P., Hu H.-W., Yang Y., He J.-Z. (2023). Bacterial Communities in the Phyllosphere Are Distinct from Those in Root and Soil, and Sensitive to Plant Species Changes in Subtropical Tree Plantations. FEMS Microbiol. Ecol..

[60] Luo X., Yan G., Wang Q., Xing Y. (2024). Community Structure, Diversity and Function of Endophytic and Soil Microorganisms in Boreal Forest. Front. Microbiol..

[61] Mahmoudi M., Almario J., Lutap K., Nieselt K., Kemen E. (2024). Microbial Communities Living inside Plant Leaves or on the Leaf Surface Are Differently Shaped by Environmental Cues. ISME Commun..

[62] Hernández-Canseco J., Bautista-Cruz A., Rincón-Enríquez G., García-Sánchez E., Aquino-Bolaños T. (2025). First Report of Drought-*Tolerant halobacteria* Associated with Agave Potatorum Zucc. Agronomy.

[63] Ujvári G., Grassi A., Avio L., Pagliarani I., Cristani C., Giovannetti M., Agnolucci M., Turrini A. (2025). Root Endophytic Bacterial Communities Are Shaped by the Specific Microbiota Associated to Mycorrhizal Symbionts. Plant Soil.

[64] Bockheim J.G., Hartemink A.E., Huang J. (2023). Sandy Soils of the World: Taxonomy, Geography, and Soil Conditions. Sandy Soils.

[65] De Holanda S.F., Vargas L.K., Granada C.E. (2023). Challenges for Sustainable Production in Sandy Soils: A Review. Environ. Dev. Sustain..

[66] Minhal F., Ma’as A., Hanudin E., Sudira P. (2020). Improvement of the Chemical Properties and Buffering Capacity of Coastal Sandy Soil as Affected by Clays and Organic By-Product Application. Soil Water Res..

[67] Ciric V., Prekop N., Seremesic S., Vojnov B., Pejic B., Radovanovic D., Marinkovic D. (2023). The Implication of Cation Exchange Capacity (Cec) Assessment for Soil Quality Management and Improvement. Agric. For..

[68] Athulya B.M., Priya G., Rani B., Aparna B., Nishan M.A. (2023). Assessment of Soil Quality Index in the Southern Coastal Sandy Soils of Kerala, India. Int. J. Environ. Clim. Change.

[69] Tripathi B.M., Stegen J.C., Kim M., Dong K., Adams J.M., Lee Y.K. (2018). Soil pH Mediates the Balance between Stochastic and Deterministic Assembly of Bacteria. ISME J..

[70] Tian J., He N., Hale L., Niu S., Yu G., Liu Y., Blagodatskaya E., Kuzyakov Y., Gao Q., Zhou J. (2018). Soil Organic Matter Availability and Climate Drive Latitudinal Patterns in Bacterial Diversity from Tropical to Cold Temperate Forests. Funct. Ecol..

[71] Lauber C.L., Hamady M., Knight R., Fierer N. (2009). Pyrosequencing-Based Assessment of Soil pH as a Predictor of Soil Bacterial Community Structure at the Continental Scale. Appl. Environ. Microbiol..

[72] Shi Y., Li Y., Yang T., Chu H. (2021). Threshold Effects of Soil pH on Microbial Co-Occurrence Structure in Acidic and Alkaline Arable Lands. Sci. Total Environ..

[73] Negi R., Sharma B., Kumar S., Chaubey K.K., Kaur T., Devi R., Yadav A., Kour D., Yadav A.N. (2024). Plant Endophytes: Unveiling Hidden Applications toward Agro-Environment Sustainability. Folia Microbiol..

[74] Zhang Q., White J.F. (2021). Bioprospecting Desert Plants for Endophytic and Biostimulant Microbes: A Strategy for Enhancing Agricultural Production in a Hotter, Drier Future. Biology.

[75] Kumar A., Chauhan P., Kumar A., Pooja, Mishra T., Padiyal A., Walia Y., Dhir S., Pandey A.K. (2025). Latest Progress (2020–2024) in Bacterial Endophyte Research with Special Reference to Plant Disease Management: Achievements and Challenges. Discov. Plants.

[76] Fanin N., Kardol P., Farrell M., Nilsson M.-C., Gundale M.J., Wardle D.A. (2019). The Ratio of Gram-Positive to Gram-Negative Bacterial PLFA Markers as an Indicator of Carbon Availability in Organic Soils. Soil Biol. Biochem..

[77] Mousa W.K., Abu-Izneid T., Salah-Tantawy A. (2024). High-Throughput Sequencing Reveals the Structure and Metabolic Resilience of Desert Microbiome Confronting Climate Change. Front. Plant Sci..

[78] Rahman S., Ahmad M., Aziz M.A., Alrayssi T.I., Mohammad A.S., Alothman R., Masmoudi K. (2025). Exploring the Bacterial Communities in Date Palm Roots in Saline versus Non-Saline Environment. BMC Plant Biol..

[79] Sen A., Saji J., Faseela P., Zhang C., Mohanan S., Xia Y. (2026). Exploring the Functional Roles of Endophytic Bacteria in Plant Stress Tolerance for Sustainable Agriculture: Diversity, Mechanisms, Applications, and Challenges. Plants.

[80] Eke P., Kumar A., Sahu K.P., Wakam L.N., Sheoran N., Ashajyothi M., Patel A., Fekam F.B. (2019). Endophytic Bacteria of Desert Cactus (*Euphorbia trigonas* Mill) Confer Drought Tolerance and Induce Growth Promotion in Tomato (*Solanum lycopersicum* L.). Microbiol. Res..

[81] Yoon H., Lee H.H., Noh H.S., Lee S.-J. (2024). Identification of Genus Deinococcus Strains by PCR Detection Using the gyrB Gene and Its Extension to Bacteria Domain. J. Microbiol. Methods.

[82] Zhao Y., Xie J., Yu S., Wu Q., Wang Z., Shang Y., Wang Z., Zhang J., Zhai H., Huang Z. (2025). A Novel Method of Species-Specific Molecular Target Mining and Accurate Discrimination of *Bacillus cereus* Sensu Lato. Int. J. Food Microbiol..

[83] Patiño-Navarrete R., Sanchis V. (2017). Evolutionary Processes and Environmental Factors Underlying the Genetic Diversity and Lifestyles of Bacillus Cereus Group Bacteria. Res. Microbiol..

[84] Liu Y., Lai Q., Göker M., Meier-Kolthoff J.P., Wang M., Sun Y., Wang L., Shao Z. (2015). Genomic Insights into the Taxonomic Status of the Bacillus Cereus Group. Sci. Rep..

[85] Schumann P., Pukall R. (2013). The Discriminatory Power of Ribotyping as Automatable Technique for Differentiation of Bacteria. Syst. Appl. Microbiol..

[86] Yamada S., Ohashi E., Agata N., Venkateswaran K. (1999). Cloning and Nucleotide Sequence Analysis of gyrB of *Bacillus cereus*, *B. thuringiensis*, *B. mycoides*, and *B. anthracis* and Their Application to the Detection of *B. cereus* in Rice. Appl. Environ. Microbiol..

[87] Carroll L.M., Cheng R.A., Wiedmann M., Kovac J. (2022). Keeping up with the Bacillus Cereus Group: Taxonomy through the Genomics Era and Beyond. Crit. Rev. Food Sci. Nutr..

[88] Bavykin S.G., Lysov Y.P., Zakhariev V., Kelly J.J., Jackman J., Stahl D.A., Cherni A. (2004). Use of 16S rRNA, 23S rRNA, and gyrB Gene Sequence Analysis To Determine Phylogenetic Relationships of Bacillus Cereus Group Microorganisms. J. Clin. Microbiol..

[89] Hinnekens P., Fayad N., Gillis A., Mahillon J. (2022). Conjugation across Bacillus Cereus and Kin: A Review. Front. Microbiol..

[90] Rutkowska N., Daroch M., Marchut-Mikołajczyk O. (2025). Exploring the Diversity and Genomics of Cultivable Bacillus-Related Endophytic Bacteria from the Medicinal Plant *Galium aparine* L. Front. Microbiol..

[91] Ali M.A., Ahmed T., Ibrahim E., Rizwan M., Chong K.P., Yong J.W.H. (2024). A Review on Mechanisms and Prospects of Endophytic Bacteria in Biocontrol of Plant Pathogenic Fungi and Their Plant Growth-Promoting Activities. Heliyon.

[92] Ercole T.G., Bonotto D.R., Hungria M., Kava V.M., Galli L.V. (2025). The Role of Endophytic Bacteria in Enhancing Plant Growth and Health for Sustainable Agriculture. Antonie Van Leeuwenhoek.

[93] Lopes R., Tsui S., Gonçalves P.J.R.O., De Queiroz M.V. (2018). A Look into a Multifunctional Toolbox: Endophytic Bacillus Species Provide Broad and Underexploited Benefits for Plants. World J. Microbiol. Biotechnol..

[94] Iqbal Z., Ahmad M., Raza M.A., Hilger T., Rasche F. (2024). Phosphate-Solubilizing *Bacillus* Sp. Modulate Soil Exoenzyme Activities and Improve Wheat Growth. Microb. Ecol..

[95] Fu Y., Lin K., Cheng B., Qi L., Zhang Q., Li H., Chen X., Zhang C. (2026). Phosphate-Solubilizing *Bacillus subtilis* Y31 Promotes Cucumber Growth and Yield: Insights from Rhizosphere Microbiomics and Bacterial Genomics. Front. Microbiol..

[96] Buisset E., Soust M., Scott P.T. (2025). The Isolation of Free-Living Nitrogen-Fixing Bacteria and the Assessment of Their Potential to Enhance Plant Growth in Combination with a Commercial Biostimulant. Microbiol. Res..

[97] Renganathan P., Astorga-Eló M., Gaysina L.A., Puente E.O.R., Sainz-Hernández J.C. (2025). Nitrogen Fixation by Diazotrophs: A Sustainable Alternative to Synthetic Fertilizers in Hydroponic Cultivation. Sustainability.

[98] Khan A., Doshi H.V., Thakur M.C., Islam M.T., Rahman M., Pandey P., Jha C.K., Aeron A. (2016). Bacillus Spp.: A Prolific Siderophore Producer. Bacilli and Agrobiotechnology.

[99] Saini N., Bundela V., Singh S., Sahgal M., Singh A.V. (2024). Optimizing Siderophore Production in *Bacillus subtilis* to Enhance Seed Germination and Biocontrol Efficacy against *Alternaria triticina* and *Bipolaris sorokiniana*. J. Sci. Res. Rep..

[100] Dogsa I., Bellich B., Blaznik M., Lagatolla C., Ravenscroft N., Rizzo R., Stopar D., Cescutti P. (2024). *Bacillus subtilis* EpsA-O: A Novel Exopolysaccharide Structure Acting as an Efficient Adhesive in Biofilms. npj Biofilms Microbiomes.

[101] Bhowmik B., Afrin S., Jui A.H., Bhuiyan R.H., Rashid M.M., Miah M.A.S., Bhuiyan M.N.I. (2025). Exploring the Purification, Characterization, and Industrial Applications of Exopolysaccharide (EPS) from *Bacillus amyloliquefaciens* Strain BDIFST240014. Mol. Biol. Rep..

[102] Sánchez-León E., Huang-Lin E., Amils R., Abrusci C. (2023). Production and Characterisation of an Exopolysaccharide by *Bacillus amyloliquefaciens*: Biotechnological Applications. Polymers.

[103] Ehinmitan E., Losenge T., Mamati E., Ngumi V., Juma P., Siamalube B. (2024). BioSolutions for Green Agriculture: Unveiling the Diverse Roles of Plant Growth-Promoting Rhizobacteria. Int. J. Microbiol..

[104] Saberi Riseh R., Vatankhah M., Hassanisaadi M., Barka E.A. (2024). Unveiling the Role of Hydrolytic Enzymes from Soil Biocontrol Bacteria in Sustainable Phytopathogen Management. Front. Biosci. (Landmark Ed.).

[105] Ling L., Li Y., Jiang K., Wang Y., Luo H., Cheng W., Pang M., Feng L., Yue R., Zhou Y. (2023). Volatile Organic Compounds of *Bacillus* Spp. as an Emerging Antifungal Resource Play a Significant Role in Fruit Postharvest Disease Control. Food Biosci..

[106] Hurtado-Bautista E., Islas-Robles A., Moreno-Hagelsieb G., Olmedo-Alvarez G. (2024). Thermal Plasticity and Evolutionary Constraints in *Bacillus*: Implications for Climate Change Adaptation. Biology.

[107] Kadapure A.J., Dalbanjan N.P., Praveen Kumar S.K. (2024). Characterization of Heat, Salt, Acid, Alkaline, and Antibiotic Stress Response in Soil Isolate Bacillus Subtilis Strain PSK.A2. Int. Microbiol..

[108] Ramzan S., Shaheen M., Khurshid M., Jabeen F., Mahmood S., Sarwar A., Ahmad S. (2024). Bioprospecting and Phylogenetic Analysis of *Priestia flexa* AW3: An Industrially Significant Amylase-Producing Bacterium from Unexplored Contaminated Soil in Layyah. J. Ind. Microbiol. Biotechnol..

[109] Soto-Varela Z.E., Orozco-Sánchez C.J., Bolívar-Anillo H.J., Martínez J.M., Rodríguez N., Consuegra-Padilla N., Robledo-Meza A., Amils R. (2024). Halotolerant Endophytic Bacteria *Priestia flexa* 7BS3110 with Hg2+ Tolerance Isolated from *Avicennia germinans* in a Caribbean Mangrove from Colombia. Microorganisms.

[110] Abdelfadil M.R., Patz S., Kolb S., Ruppel S. (2024). Unveiling the Influence of Salinity on Bacterial Microbiome Assembly of Halophytes and Crops. Environ. Microbiome.

[111] Olagunju O.O., Ogunnusi T.A., Akpor O.B. (2025). Evaluation of Indole Acetic Acid and Hydrogen Cyanide Production by Plant Growth Promoting Rhizobacteria. Vegetos.

[112] Singh P., Chauhan P.K., Upadhyay S.K., Singh R.K., Dwivedi P., Wang J., Jain D., Jiang M. (2022). Mechanistic Insights and Potential Use of Siderophores Producing Microbes in Rhizosphere for Mitigation of Stress in Plants Grown in Degraded Land. Front. Microbiol..

[113] Yang Y., Wang W., Meng X., Niu B., Yang J., Chen Q. (2025). The betA/B Genes as a Key Factor in *Cronobacter sakazakii* Survival under Desiccation Stress. J. Dairy Sci..

[114] Vale P.F. (2025). *Providencia Rettgeri*. Trends Microbiol..

[115] Qin Y.-L., Liang Z.-L., Ai G.-M., Liu W.-F., Tao Y., Jiang C.-Y., Liu S.-J., Li D.-F. (2024). Heterotrophic Nitrification by *Alcaligenes faecalis* Links Organic and Inorganic Nitrogen Metabolism. ISME J..

[116] Waheed Z., Iqbal S., Irfan M., Jabeen K., Umar A., Aljowaie R.M., Almutairi S.M., Gancarz M. (2024). *Pseudochrobactrum asaccharolyticum* Mitigates Arsenic Induced Oxidative Stress of Maize Plant by Enhancing Water Status and Antioxidant Defense System. BMC Plant Biol..

[117] Aguennouz R., Aallam Y., Haddioui A., Hamdali H. (2025). Unlocking Plant Growth-Promoting Traits of Endophytic Actinobacteria Isolated from *Anacyclus pyrethrum*, an Endemic Medicinal Plant of the Aguelmam Azegza Region, Morocco. Front. Microbiol..

[118] Boukelloul I., Aouar L., Cherb N., Carvalho M.F., Oliveira R.S., Akkal S., Nieto G., Zellagui A., Necib Y. (2024). Actinobacteria Isolated from Soils of Arid Saharan Regions Display Simultaneous Antifungal and Plant Growth Promoting Activities. Curr. Microbiol..

